# Age Analysis of Status Updating System with Probabilistic Packet Preemption

**DOI:** 10.3390/e24060785

**Published:** 2022-06-02

**Authors:** Jixiang Zhang, Yinfei Xu

**Affiliations:** School of Information Science and Engineering, Southeast University, Nanjing 210096, China; zhangjx@seu.edu.cn

**Keywords:** age of information, discrete time status updating system, probabilistic preemption, probability generation function, stationary distribution

## Abstract

The age of information (AoI) metric was proposed to measure the freshness of messages obtained at the terminal node of a status updating system. In this paper, the AoI of a discrete time status updating system with probabilistic packet preemption is investigated by analyzing the steady state of a three-dimensional discrete stochastic process. We assume that the queue used in the system is $Ber/Geo/1/2^{*}/\eta$, which represents that the system size is 2 and the packet in the buffer can be preempted by a fresher packet with probability η. Instead of considering the system’s AoI separately, we use a three-dimensional state vector (n,m,l) to simultaneously track the real-time changes of the AoI, the age of a packet in the server, and the age of a packet waiting in the buffer. We give the explicit expression of the system’s average AoI and show that the average AoI of the system without packet preemption is obtained by letting η=0. When η is set to 1, the mean of the AoI of the system with a $Ber/Geo/1/2^{*}$ queue is obtained as well. Combining the results we have obtained and comparing them with corresponding average continuous AoIs, we propose a possible relationship between the average discrete AoI with the Ber/Geo/1/c queue and the average continuous AoI with the M/M/1/c queue. For each of two extreme cases where η=0 and η=1, we also determine the stationary distribution of AoI using the probability generation function (PGF) method. The relations between the average AoI and the packet preemption probability η, as well as the AoI’s distribution curves in two extreme cases, are illustrated by numerical simulations. Notice that the probabilistic packet preemption may occur, for example, in an energy harvest (EH) node of a wireless sensor network, where the packet in the buffer can be replaced only when the node collects enough energy. In particular, to exhibit the usefulness of our idea and methods and highlight the merits of considering discrete time systems, in this paper, we provide detailed discussions showing how the results about continuous AoI are derived by analyzing the corresponding discrete time system and how the discrete age analysis is generalized to the system with multiple sources. In terms of packet service process, we also propose an idea to analyze the AoI of a system when the service time distribution is arbitrary.

## 1. Introduction

The freshness of transmitted messages has attracted increased attention in the design of practical communication systems. Messages obtained by a controller in a real-time monitor system may be used to perform traffic scheduling or resource allocation, and for such applications, the system’s timeliness is crucial for the scheduler to make the right response and for precise control. The age of information (AoI) metric was proposed in [1] as the time elapsed since the generation time of the last received packet in the destination, which has been used widely in recent years to measure the packet’s freshness and characterize the timeliness of various communication networks. A simple introduction to the AoI theory can be found in [2], and in [3], the authors made a detailed summary about the analytical results of age of information, along with employing the AoI optimization in many cyber-physical applications.

### 1.1. Related Work

For a status updating system with simple queue models, such as M/M/1, M/D/1, and D/M/1 queues, the expression of average AoI was obtained in [4,5,6,7]. In particular, in [7], the authors considered a queue using Last-Come-First-Served (LCFS) discipline, and the newer packet from the source could preempt the packet currently in service. The influence of different packet management strategies on a system’s average AoI was investigated in [8,9], where only one or two packets can be stored in the system. Specifically, the average AoI of a system with three queues—that is, M/M/1/1, M/M/1/2, and M/M/1/2*—was determined. The difference between last two queues lies in whether the packet waiting in the buffer can be substituted by following packets from the source. For two cases with a system size equal to 2, it was shown that updating the waiting packet with a fresher one can always result in a lower average AoI, which is apparent because transmitting the packet with a smaller age is helpful for improving the timeliness of the information transmission systems. Apart from these, the benefit of introducing a proper packet deadline, both deterministic and random, to reduce the average age of information was proved in [10,11,12]. Controlling packet preemptions to improve the freshness of a transmitted message was discussed in [13,14,15]. The authors of [16] showed that the average AoI can be significantly improved when adding a period of waiting time before the service of a new packet begins. Assuming there are two parallel servers in the status updating system, the expressions of the average AoI were determined in [17]. A freshness-based cache updating in a parallel relay network was considered in [18]. Notice that when more than one server was present, the updating packet could reach the destination through different paths. In these situations, since a packet generated behind may be transmitted to destination via a short-delay path, it is possible that this packet arrives at the receiver before the packets generated before it. Recently, many papers have been launched considering the AoI of status updating networks with simple structures, such as the status updating system with multiple sources [19,20,21,22,23,24,25], the system with more than one hop transmission [26,27,28,29,30], and the system in which the packet transmission is assisted by a relay [31,32,33,34,35]. Recently, using the SHS method, the AoI of an arbitrarily connected network named the gossip network was discussed in [36,37]. For each of the above systems, the average performance of the AoI was characterized, and even some properties of the AoI’s distribution were obtained in certain papers. For example, for the age on a line network of preemptive memoryless servers, in [38], the author proved that the age at a node is identical in distribution to the sum of independent exponential service times by calculating the Moment Generation Function (MGF) of the defined age vector. In [39,40], the distribution of AoI was studied in a wireless networked control system with two-hop packet transmission. The authors devised the problem of minimizing the tail of the AoI distribution with respect to the sampling rate under a First-Come First-Serve (FCFS) queuing discipline. In [41], for the phase-type (PH-type) interarrival time or packet service time, the authors numerically obtained the exact distribution of the (peak) age of information for the system with PH/PH/1/1 and M/PH/1/2 queues. Within the paper, they used the sample path arguments and the theory of Markov Fluid Queues (MFQ). Except for the works we mentioned above which focus on obtaining analytical results of the AoI for status updating systems with various queue models, even more papers have been published in which the authors considered designing optimal systems under different timeliness requirements, such as in [42,43,44,45,46,47,48,49,50,51]. In such problems, usually the age of information is used as a freshness metric and is studied as the optimization objective.

### 1.2. Discussion of Existing Methods

As far as we know, at least three methods have been proposed to analyze the AoI of a continuous time status updating system. The first one is the method based on the graph of the AoI stochastic process, which was given in [2]. The time average AoI is obtained by calculating the area below the sample path of the AoI process. Using the common assumption that the age process is ergodic, this time average AoI converges to the AoI’s mean as the observation time tends to infinity. It shows that the average AoI of a status updating system is determined by

(1) $$E\left[\Delta \right]=\frac{E\left[YT\right]+E\left[Y^{2}\right]/2}{E[Y]}$$

when the packet arrival process and the distribution of service time are specified, in which the notation *Y* denotes the interarrival time between two successive updating packets, and *T* represents the packet system time. Secondly, in [6], the authors illustrated the usage of the Stochastic Hybrid System (SHS) approach to the analysis of system’s stationary AoI. They employed a continuous state vector to track the real-time age of the updating packets from the source and described all the possible state vector transfers under the system’s random dynamics—for example, if a new packet arrives, whether the packet service is completed. Then, the steady state of the multiple-dimensional continuous time Markov process was characterized by a group of differential equations, and the first few of the AoI’s moments could be obtained using the theory of SHS [52]. This method was used later to determine the average AoI of more general systems, including the system with multiple sources, packet preemption, and even stochastic energy harvesting at certain system nodes. The last method was introduced in [5], where the authors proposed a novel description of the AoI process and characterized its sample paths using a new point process. They proved that the stationary distribution of the AoI can be represented in terms of the distributions of the system’s delay and the peak AoI. From this point of view, large numbers of analytical formulas about the AoI’s stationary distribution were obtained (in the form of its Laplace Stieljes Transform (LST)) for single-server systems. In addition, we found that the same method has been used to consider the distribution of discrete time (peak) AoI in [53,54], where the *z*-transform of the (peak) AoI’s distribution was derived for the system with some discrete queues.

Although plenty of results have been obtained using the methods mentioned above, interested readers may find that most of the results are heavily dependent on the assumptions that the packet arrivals form a Poisson process and the service time distribution are exponential, especially for the SHS method. The memoryless property of both interarrival time distribution and the distribution of packet service time dramatically simplifies the age analysis of the considered status updating system. So far, the first method based on the graphical argument of the AoI process is used only to calculate the AoI’s mean, but it seems that the theory of Level Crossing in [55] may be useful when considering the AoI’s distribution from the sample paths themselves. The level crossing method has been used to derive the steady-state probability density function of queue waiting in several variants of the M/G/1 queue. It is worth trying to determine whether this theory can be used to find the stationary distribution of continuous AoI. Using the SHS method, similarly, only the first few of the AoI’s moments can be calculated. In order to obtain the distribution property of system’s AoI, one has to solve the system of differential equations, which is extremely hard in general. At last, in [5], the authors pointed out that the general formula they proposed holds sample-path-wise, regardless of the service discipline or the distributions of interarrival and packet service times; however, the results they obtained are not straight-forward, as they only derived the LST of the AoI’s stationary distribution, while computing the explicit expression of this distribution is also a hard problem due to the difficulty of computing the inverse of the LST. On the other hand, it is unknown if the method and the obtained formula can be generalized to more general status updating systems, not just for the system with a single server.

In the following part, we introduce the idea and methods to analyze the AoI of discrete time status updating systems and talk about their merits compared with those ways dealing with continuous time age of information. By an explicit example, we show how the results of continuous AoI can be obtained by considering the corresponding discrete time systems.

### 1.3. Analysis of Discrete Time AoI: Idea and Methods

We propose the idea and methods to characterize the steady state AoI of a discrete time status updating system, in which the packet arrivals, the packet service, and AoI declines are considered in discrete time slots. Although there are not many, there are still some works analyzing the AoI of a discrete system with different queue models. To our best knowledge, the analysis of discrete AoI was proposed for the first time in [56]. Using the proof techniques and tools developed to analyze continuous AoI, the authors obtained the average (peak) AoI of a Ber/G/1 and G/G/∞ queue modeled discrete time status updating system. The notation “Ber” represents that the packet arrival or the service of the packet forms a Bernoulli stochastic process; equivalently, in each time slot, a packet arrives (or the packet service is completed), which is independent and occurs with an identical probability. Later, using the similar description of the age process’s sample path as in [5], in [53,54] the expression of the discrete AoI’s distribution was obtained for the system with a First-Come First-Served (FCFS) queue, the preemptive Last-Come First-Served (LCFS) queue, and the bufferless status updating system. Discrete time systems with multiple sources are considered in [57]. Under the assumption of Bernoulli packet arrivals and a common general discrete phase-type service time distribution across all the sources, the authors obtained the exact per-source distributions of AoI and peak AoI in matrix-geometric form for three different queueing disciplines, i.e., nonpreemptive bufferless, preemptive bufferless, and nonpreemptive single buffer with replacement.

In our work [58], we obtain the explicit formula of average discrete AoI, ${\bar{\Delta }}_{Ber/Geo/1/1}$ for a bufferless status updating system (actually, the service time distribution in [58] is arbitrary) by defining a two-dimensional age process which characterizes the AoI at the destination and the age of packet in service as a whole. The idea we proposed in [58] can be regarded as the discretization of the SHS method, which is shown to be equally powerful and more flexible when applied to more general systems. We describe all the possible state transfers for every initial state vector and then establish the stationary equations of the defined two-dimensional discrete age process. These equations are solved completely in [58]; thus, the distribution of AoI can be determined explicitly as one of the marginal distributions of the two-dimensional age process’s stationary distribution. Given the AoI’s distribution, the mean, the variance, and the tail probabilities of the AoI can be easily calculated. The idea of constituting multiple-dimensional age processes is then used in [59] to obtain the mean and the distribution of the infinite size state updating system. In [60], the distributions of the AoI of a system with Ber/Geo/1/1, Ber/Geo/1/2, and Ber/Geo/1/2* queues are derived explicitly using the method of solving equations. In this paper, the AoIs of a system with Ber/Geo/1/2 and Ber/Geo/1/2* queues are considered simultaneously, which are connected together by the probabilistic packet preemption in the system’s buffer. In addition, in order to avoid the tedious calculation required to solve the stationary equations and calculate the marginal distribution, we define the Probability Generation Function (PGF) of the multiple-dimensional stationary distribution, from which both the AoI’s mean and its stationary distribution can be obtained effectively. For the system’s average AoI, in Table 1, we list the results we have obtained about the discrete AoI and the corresponding expressions of the continuous system’s average AoI. The average AoI ${\bar{\Delta }}_{Ber/Geo/1/1}$ was obtained in [58], and the other two expressions will be derived in the current paper. Apart from the AoI’s mean, we also determine the distribution of the discrete AoI $\Delta_{Ber/Geo/1/2}$ and $\Delta_{Ber/Geo/1/2^{*}}$ by writing the PGF as the power series.

As mentioned above, one can see the similarity between our idea and the SHS method, and one may mistakenly think that we simply change the continuous time into discrete time slots. The power of combining multiple-dimensional state vector descriptions with the PGF method may be underestimated due to the simple assumptions used in the current paper—that is, the packet arrivals form a Bernoulli process and the packet service time is geometrically distributed. It is known that in order to obtain the complete statistical information, not just the mean of the stationary AoI by the method of SHS, one has to solve a group of differential equations, which may be possible for some systems with simple queues but generally is impossible. In addition, the usage of SHS analysis is heavily restricted because it requires that both the packet arrival process and the packet service process are memoryless, i.e., the interarrival time and the packet service time have to be i.i.d. exponential random variables. In the following, we explain the merits of considering a discrete time system in two aspects.

(1) Calculation: reducing the complexity

Observing that when all the state transitions are described in discrete time slots, the stationary equations characterizing the steady state of the defined age process become a set of linear equations, which is more likely to be solved compared with those differential equations, we show in this paper that these linear equations can be dealt with using the PGF method even more easily and more effectively. In our another work, we have determined the explicit expression of average AoI and the corresponding AoI’s distribution assuming the Ber/Geo/1/c queue is used in the status updating system, where the system’s size *c* can be arbitrary. For the cases c=3 and 4, we obtain that

(2) $${\bar{\Delta }}_{Ber/Geo/1/3}=\frac{1}{\gamma }\left(\left(1-\gamma \right)+\frac{1}{\rho_{d}}+\frac{\rho_{d}^{2}\left(1-\gamma^{2}\right)+3\rho_{d}^{3}\left(1-5\gamma /3+\gamma^{2}/3\right)}{1+\rho_{d}\left(1-3\gamma \right)+\rho_{d}^{2}\left(1-3\gamma +3\gamma^{2}\right)+\rho_{d}^{3}{\left(1-\gamma \right)}^{3}}\right)$$

and

(3) $$\begin{array}{cc} {\bar{\Delta }}_{Ber/Geo/1/4} & =\frac{1}{\gamma }(\left(1-\gamma \right)+\frac{1}{\rho_{d}}\\ \; & \;+\frac{\rho_{d}^{2}\left(1-\gamma \right)+2\rho_{d}^{3}\left(1-\gamma \right)\left(1-2\gamma \right)+4\rho_{d}^{4}\left(1-\gamma \right)\left(1-11\gamma /4+9\gamma^{2}/4-\gamma^{3}/4\right)}{1+\rho_{d}\left(1-4\gamma \right)+\rho_{d}^{2}\left(1-4\gamma +6\gamma^{2}\right)+\rho_{d}^{3}\left(1-4\gamma +6\gamma^{2}-4\gamma^{3}\right)+\rho_{d}^{4}{\left(1-\gamma \right)}^{4}}) \end{array}$$

Although we have not mentioned this yet, the readers should find that those expressions of average continuous and average discrete AoI given in Table 1 are quite similar. We propose the following possible relationship:

(4) $$\mu \cdot {\bar{\Delta }}_{M/M/1/c}={\left.\gamma \cdot {\bar{\Delta }}_{Ber/Geo/1/c}\right|}_{\gamma =0},\;\mathrm{then}\;\mathrm{replacing}\;\rho_{d}\;\mathrm{with}\;\rho$$

The relation (4) holds at least for c=1, c=2, and $c=2^{*}$. Note that the relation (4) is given only by observation, and it is not easy to prove that (4) is applicable in general situations, because the average continuous AoI ${\bar{\Delta }}_{M/M/1/c}$ is temporarily unknown. If Equation (4) is fortunately applicable in general, which we hope, then from expressions (2) and (3), immediately we have

(5) $${\bar{\Delta }}_{M/M/1/3}=\frac{1}{\mu }\left(1+\frac{1}{\rho }+\frac{\rho^{2}+3\rho^{3}}{1+\rho +\rho^{2}+\rho^{3}}\right)$$

and

(6) $${\bar{\Delta }}_{M/M/1/4}=\frac{1}{\mu }\left(1+\frac{1}{\rho }+\frac{\rho^{2}+2\rho^{3}+4\rho^{4}}{1+\rho +\rho^{2}+\rho^{3}+\rho^{4}}\right)$$

Notice that the average continuous AoIs (5) and (6) are not derived using any of the three methods we discussed earlier—that is, the method based on the sample path of the AoI process, the SHS, and the method proposed in [5,54]. On the contrary, we first characterize the stationary AoI of the corresponding discrete time system and then obtain the expression of the continuous AoI’s mean through relationship (4). There is no doubt that the formulas of ${\bar{\Delta }}_{M/M/1/3}$ and ${\bar{\Delta }}_{M/M/1/4}$ can be obtained using AoI’s SHS analysis; however, the general formula of AoI’s mean, i.e., ${\bar{\Delta }}_{M/M/1/c}$ for arbitrary size *c* is temporarily unknown. Furthermore, the stationary distribution of discrete AoI can also be determined explicitly from the PGF defined for the considered system, while the distribution properties of the continuous AoI cannot be revealed easily through either the graphical method or the AoI’s SHS analysis. Although it is not possible to accurately reprint the continuous AoI’s distribution in every position using the discrete approximation, the difference between them can be reasonably small when the length of the time slot is short enough. In the current paper, we determine the distribution expressions of discrete AoI for the system with Ber/Geo/1/2 and $Ber/Geo/1/2^{*}$ queues. Unlike in [5,54], these expressions are straight-forward and not expressed in the form of other transformations.

According to vabove discussions, from the perspective of deriving the average AoI or obtaining the AoI’s distribution, considering the status updating system in the discrete time model is of great significance. To a certain extent, we can even conclude that our method is stronger since more specific results about AoI have been obtained.

(2) Generalization: In terms of system structure and service time distribution

**Figure 1:**
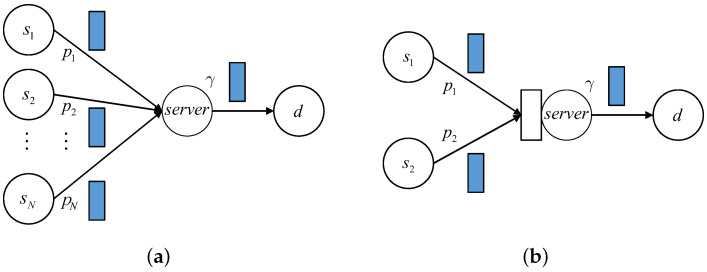
(**a**) Status updating system with multiple sources and bufferless server. (**b**) Status updating system with two sources and a size 1 buffer.

Recently, using the SHS method, the age analysis has been generalized to the status updating networks with a simple structure, especially the system with multiple sources. In this part, we briefly explain how the discrete age of information is characterized in the multiple-source bufferless system and the two-source system equipped with a size 1 buffer. The system models are depicted in Figure 1.

Specifically, we assume the packets arrive at the beginning of one time slot; whether the packet service is completed is determined at the end of the time slot. Since the system’s random dynamics are considered in time slots, it is possible that more than one packet arrives to the server (buffer) from different sources in a time slot. The server has to choose one of these and discard the other packets if the system does not have a buffer. This packet collision problem can be solved by assigning priorities to the packets from different sources; then, the packet with the highest priority is selected and put into the server.

In the bufferless system given in the first picture of Figure 1, let $r_{i}$ be the priority of source $s_{i}$, 1≤i≤N, and assume $r_{1}>r_{2}>\cdots >r_{N}$—that is, the priority of source $s_{i}$ is over that of $s_{j}$ if i<j. In each time slot, source $s_{i}$ generates a new packet with probability $p_{i}$, and the packet generation process is independent of all other sources. Actually, this situation is exactly the generalization of our work in [58] when the status updating system has multiple independent sources. For the given i∈[1,N], it shows that the AoI process corresponding to source $s_{i}$ can be analyzed separately and is thus similar to work [58], showing that a two-dimensional state vector $\left(n_{i},m_{i}\right)$ is sufficient to track the real-time changes of $AoI_{i}$ and the age of the packet in the server from source $s_{i}$. In this system, we observe that it does not matter whether the service of the packet from $s_{i}$ can be preempted by other packets with higher priorities. The state vector transfers from every $\left(n_{i},m_{i}\right)$ can be described as in [58], but the transition probabilities need to be modified. For example, for $n_{i}>m_{i}\geq 1$, we have

(7) $$State\;vector\;at\;next\;time\;slot=\left\{\begin{array}{cc} \left(n_{i}+1,m_{i}+1\right) & \mathrm{the}\;\mathrm{packet}\;\mathrm{service}\;\mathrm{is}\;\mathrm{not}\;\mathrm{completed},\\ \left(m_{i}+1,0\right) & \mathrm{the}\;\mathrm{service}\;\mathrm{of}\;\mathrm{the}\;\mathrm{packet}\;\mathrm{is}\;\mathrm{over}. \end{array}\right.$$

if the service process cannot be preempted. In contrast, it can be decided that

(8) $$State\;vector\;at\;next\;time\;slot=\left\{\begin{array}{cc} \left(n_{i}+1,m_{i}+1\right) & \mathrm{no}\;\mathrm{packets}\;\mathrm{of}\;\mathrm{higher}\;\mathrm{priorities}\;\mathrm{arrive},\;\mathrm{the}\;\mathrm{service}\;\mathrm{is}\;\mathrm{not}\;\mathrm{over},\\ \left(n_{i}+1,0\right) & \mathrm{one}\;\mathrm{packet}\;\mathrm{with}\;\mathrm{higher}\;\mathrm{priority}\;\mathrm{comes},\\ \left(m_{i}+1,0\right) & \mathrm{no}\;\mathrm{packets}\;\mathrm{with}\;\mathrm{higher}\;\mathrm{priorities}\;\mathrm{arrive},\;\mathrm{the}\;\mathrm{service}\;\mathrm{is}\;\mathrm{over}. \end{array}\right.$$

when packet service preemption is allowable. After all the state transfers are described and their transition probabilities are determined, we can obtain the stationary equations, which can be solved completely as in [58] or by using the PGF method as in this paper. Like [58], the service time distribution in this case can be arbitrary.

Although there are multiple sources, it can be seen that the age analysis of each source is easy when the status updating system has no buffer. Notice that in this case, no queue is formed before the server; thus, there is no chance that the packets from different sources are combined. As a result, the packets from every source are totally divided, and the AoI of each source can be analyzed separately. The situation is much more difficult if the system has a buffer. As an example, we consider the AoI of each source of a two-source system, which is depicted in the second picture of Figure 1.

We can define a six-dimensional state vector $\left(n_{1},n_{2},m_{1},m_{2},l_{1},l_{2}\right)$ to describe the AoI of two sources simultaneously, where the state components represent the values of two AoIs at the destination, the age of a packet in the server, and the age of a packet in the system’s buffer. In every position of the system, apart from the ”age”, it is necessary to indicate which source the packet comes from. Therefore, a three-dimensional state vector (n,m,l) that does not include this information is not sufficient. Notice that at any time, at most one of $m_{1}$ and $m_{2}$ are non-zero. This is the same for the parameters $l_{1}$ and $l_{2}$. When there is a buffer in front of the server, apparently a queue is formed if a packet arrives and finds that the server is currently busy. Each one of two packets in the system (one is in the server and the other is in the buffer) may come from source $s_{1}$ or $s_{2}$. Of course, these two packets may belong to different sources. Although the problem becomes complex, theoretically, all the state transfers of every initial six-dimensional state vector can be determined explicitly, since the randomness that causes the state vector transfers are limited to random packet arrival, the service of the packet, and the additional packet preemption. Then, according to the balance of probabilities in the steady state, the stationary equations are established; this solves the first half of the AoI analysis. Details of the latter half—that is, deriving the average AoI from the group of stationary equations—can be found in the procedures in this article.

We find that in [61], the authors obtained the average continuous AoI for the same two-source status updating system in Figure 1b using the SHS method. They added another assumption that the packet in the server and the packet in the buffer must belong to different sources in their second and the third considered situation and named the policies “source-aware packet management”. As we have mentioned above, although the packets from two sources are still combined, after adding this restriction, the complexity of the problem has been greatly reduced.

In fact, the state vector defined for a discrete time system has a very clear physical meaning. For the status updating system with the FCFS queue, the first parameter denotes the AoI and the other state components represent the ages of packets in the server and in the buffer of the system. Thus, a (c+1)-dimensional state vector is needed if the size of the system is equal to *c*. Compared with analyzing the AoI of discrete systems, in the SHS method, the defined state vector is sometimes easier, such as in the system with multi-sources. In [61], in order to characterize the AoI of one source in a two-source system, the authors used a four-dimensional state vector $\left[x_{0}\left(t\right),x_{1}\left(t\right),x_{2}\left(t\right),x_{3}\left(t\right)\right]$ that describes the evolutions of AoI when different random events occur. As mentioned before, we use the six-dimensional state vector $\left(n_{1},n_{2},m_{1},m_{2},l_{1},l_{2}\right)$ describing the random changes of both source 1 and source 2. The parameter $n_{1}$ or $n_{2}$ can also be deleted if only one of two sources are analyzed. In our proposed method, we show the correspondence between the dimension of the state vector and the size of the discrete system; this may not be a unique way to define the discrete state vector. Although considering the AoI of the discrete time system has higher computational complexity, the biggest advantage of discrete AoI analysis is that it can obtain the stationary distribution of the AoI.

Except the simple status updating networks given in Figure 1, we have also obtained the average discrete AoI for a status updating system with two-stage service, where for simplicity, in front of each server, no buffer is equipped. For the system with two parallel servers, the age analysis is more difficult, since some packets may become “ineffective” if one packet is generated later but arrives to the destination earlier. Some policies need to be identified to deal with these packets—for instance, deleting the packet directly once it becomes ineffective. If nothing is done, when an ineffective packet is obtained at the receiver, the value of AoI will not be reduced.

Another direction of generalization we shall talk about is the distribution of packet service time (while the packet arrival process is still Bernoulli). Now, taking the size 2 status updating system as an example, we explain how the service time distribution is relaxed to be an arbitrary distribution. Using a three-dimensional state vector (n,m,l), we can fully describe the random dynamics including the AoI at the receiver and the age of two packets in the system if both the packet interarrival time and the service time have memoryless properties. In each time slot, the changes of the AoI’s value and the packet ages depend on random packet arrival, which is memoryless and independent, and whether the packet service is over. When the service time distribution is arbitrary, the probability that the service is completed in one time slot is related to the time this packet has experienced in the server. Let *S* be the random variable of service time, and we represent the general distribution as

(9) $$\mathrm{Pr}\left\{S=i\right\}=q_{i}\;\left(i\geq 1\right)$$

We assume that, before the current time slot, the packet has stayed in server for *j* time slots; then, the probabilities that determine the state vector transfers should be the following two conditional probabilities:

(10) $$\mathrm{Pr}\left\{S=j+1|S>j\right\}\;\mathrm{and}\;\mathrm{Pr}\left\{S>j+1|S>j\right\}$$

Therefore, if we have knowledge about this passed service time *j*, as before, all the state transfers of state vector (n,m,l) can be completely described and the age analysis becomes feasible. Since no one of the three parameters *n*, *m*, and *l* can provide this information, it is natural to introduce an extra component, say *k*, to denote the service time that the packet has consumed and constitute the four-dimensional state vector (n,m,l,k). In this way, the possible state transfers of this four-dimensional state vector can be described and the transition probabilities can also be determined. For example, let the initial state vector be (n,m,l,k)—we have the state transfers and transition probabilities as

(11) $$Next\;state\;vector=\left\{\begin{array}{cc} \left(n+1,m+1,l+1,k+1\right) & \mathrm{the}\;\mathrm{service}\;\mathrm{is}\;\mathrm{not}\;\mathrm{over}\;\mathrm{with}\;\mathrm{prob}.\;1-q_{k+1}/\sum_{i=k+1}^{\infty }q_{i}\\ \left(m+1,l+1,0,0\right) & \mathrm{the}\;\mathrm{service}\;\mathrm{completes}\;\mathrm{with}\;\mathrm{prob}.\;q_{k+1}/\sum_{i=k+1}^{\infty }q_{i} \end{array}\right.$$

where we assume the queue discipline is FCFS and there is no packet preemption. We show that the four parameters *n*, *m*, *l*, and *k* satisfy the relationships n>m>l≥0 and n>m≥k≥0. The first one holds because *n*, *m*, and *l* are three ages of packets generated in chronological order, and n>m≥k is satisfied since the packet system time *m* must be larger than or equal to the service time of the packet, which is denoted by *k*. These relations determine which vectors are qualified state vectors. Although we show that the state transfers can be analyzed and the group of stationary equations can be determined by considering the balance of those stationary probabilities; however, it can be expected that solving these equations is not easy. Since the service time probabilities $q_{i}$s are arbitrary, the expression of the average AoI, as we can determine in later work, will not be closed-formed. It is also important to note that the PGF method cannot be used when the service time distribution is not geometric, because the transition probabilities is no longer the same for different state vectors and thus cannot be the common factor.

Summarizing the above discussions, we have proved that on the basis of original memoryless status updating system, by introducing an extra component to denote the time the packet has consumed in the server, the age analysis becomes feasible for the situation where the packet service time is arbitrarily distributed. Although it may be difficult to obtain the expressions of the system’s average AoI, the idea is still applicable when we generalize the size 2 system to a status updating system with arbitrary size *c*. In one of our works, we have shown that for a size *c* discrete time status updating system with Bernoulli packet arrivals and geometrically distributed service time, in order to fully characterize the real time transfers of the system’s AoI and all the packet ages, a (c+1) dimensional state vector $\left(n,m_{1},\cdots ,m_{c}\right)$ should be defined. By adding an extra state component *k* that records the service time the packet has experienced in the server, according to previous discussions, the age analysis can be generalized to a size *c* status updating system whose service time distribution is arbitrary (at least we can establish all the stationary equations).

We have to attribute the above idea to [62], in which the authors considered the timely transmission of the updates over an erasure channel. They assume that each update consists of *k* symbols and the symbol erasure in each time slot is an i.i.d. Bernoulli process. The aim of [62] is to design an optimal online transmission scheme to minimize the time average AoI, and the problem is formulated as a Markov Decision Process (MDP). Although the optimization of AoI is not our interest, the state tuple $\left(\delta_{t},d_{t},l_{t}\right)$ defined in Section 2. A is very enlightening, based on which the transmission policy at the next time slot is determined. At the *t*-th time slot, the notation $\delta_{t}$ denotes the value of AoI, $d_{t}$ is the age of the next update, i.e., the packet at the head of the queue, and $l_{t}$ records the number of symbols that has been obtained successfully up to this time slot—these symbols belong to the update that is transmitted currently. A similar timely source coding problem was also discussed in [63], in which the authors also pointed out that the length of the encoded update is equivalent to the service time of the update, and their considered system behaves as a discrete time Geo/G/1 queue (we use the notation Ber/G/1). Therefore, the role of $l_{t}$ in [62] can be regarded (or redefined) as the service time that the current update has consumed. By adding this knowledge, the distribution of the source in these papers and the service time distribution in the discrete time status updating system which we study in this part can be arbitrary.

In previous paragraphs, we explain the idea and methods used to study the AoI of discrete time status updating systems. We have shown how the discrete AoI is characterized for the basic system, the system with multiple sources, and the system whose service time distribution is arbitrary. As part of AoI theory, we believe that discrete AoI deserves more attention, and it is meaningful to establish analytical results including the AoI’s mean and its distribution for more general systems. In particular, the proposed possible relationship in (4) shows that discussing discrete AoI not only has independent theoretical significance but also helps to determine certain results about continuous AoI. If one problem is difficult in the continuous time model, it is a choice to consider it in discrete time settings.

### 1.4. The Work in the Current Paper

We have discussed numerous topics of discrete AoI in the previous subsection, and it is inappropriate to consider all the issues in one article. In this paper, we focus on the stationary AoI of a discrete time system with a Ber/Geo/1/2 and $Ber/Geo/1/2^{*}$ queue and discuss both in a single model. We assume the packet in the buffer can be probabilistically preempted by the fresher packets from the source and define the queue model in this scenario as $Ber/Geo/1/2^{*}/\eta$, where η is the preemption probability. In the literature of AoI, the probabilistic packet preemption (replacement) has been studied in [64]. In [65], the probabilistic preemption was considered in the scenario where a CPU is used frequently to deal with the unpredictable tasks. Then, for the case of η=0, the queue model of the system reduces to Ber/Geo/1/2, while when η is equal to 1, the status updating system with $Ber/Geo/1/2^{*}$ queue is obtained. For the general case, we derive the explicit expression of the system’s average AoI. By writing the defined PGF as the power series, for two extreme cases of η=0 and η=1, the distribution expressions of two discrete AoIs are determined as well.

The rest of the paper is organized as follows. In Section 2, we describe the model of a discrete time status updating system with probabilistic packet preemption. The stationary distribution and the mean of the system’s AoI are also defined. By analyzing the steady state of a three-dimensional stochastic age process, in Section 3, we obtain the explicit formula of the average AoI under general preemption probability using the probability generation function (PGF) method. In Section 4, let η=0 and η=1, and we determine the average AoIs ${\bar{\Delta }}_{Ber/Geo/1/2}$ and ${\bar{\Delta }}_{Ber/Geo/1/2^{*}}$ from the general expression derived previously in Section 3. Furthermore, in order to obtain the stationary distribution of two discrete AoIs, we write the PGF as power series. Then, the coefficient before $x^{n}$ gives the probability that the AoI takes value *n* for each n≥1. Numerical results are placed in Section 5. For the general case, we illustrate the relationships between the average AoI and η and the traffic intensity $\rho_{d}$, respectively. In addition, the mean and the cumulative probabilities of three discrete AoIs including $\Delta_{Ber/Geo/1/1}$, $\Delta_{Ber/Geo/1/2}$, and $\Delta_{Ber/Geo/1/2^{*}}$ are depicted. These average discrete AoIs and their corresponding average continuous AoIs are also numerically compared in Section 5. Finally, we conclude the paper in Section 6.

## 2. System Model and Problem Formulation

**Figure 2:**
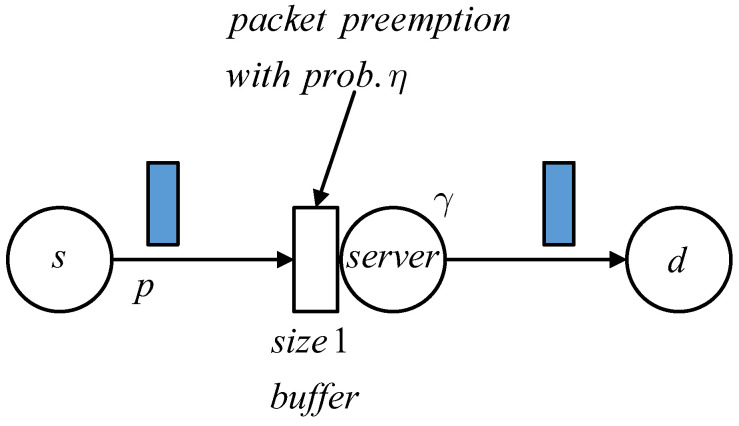
The model of the discrete time status updating system with probabilistic packet preemption in the system’s buffer.

We depict the model of the status updating system which uses the $Ber/Geo/1/2^{*}/\eta$ queue in Figure 2, in which the packet in the system’s buffer can be preempted by a fresher packet from the source *s* with probability η. The packet arrivals to the transmitter are assumed to form a Bernoulli stochastic process—that is, in each time slot, a new packet comes with an identical probability, which we denote by *p*. Packet service time follows the geometric distribution with intensity γ. The updated packet generated at *s* is transmitted to the destination *d* through the transmitter, in which a random period of time is consumed. The age of information (AoI) at *d* is defined as the time elapsed since the generation time of the last obtained packet. Within the time when no packet is received, the value of AoI increases by 1 after each time slot ends. Every time a packet passes the transmitter and arrives to *d*, the AoI will be reduced to the system time of the obtained packet, which is actually equal to the instantaneous age of this packet.

Let a(k) be the value of AoI in the *k*th time slot. The AoI at the next time slot, a(k+1), is determined by

(12) $$a\left(k+1\right)=\left\{\begin{array}{cc} a(k)+1 & \mathrm{if}\;\mathrm{no}\;\mathrm{packet}\;\mathrm{is}\;\mathrm{obtained},\\ a\left(k\right)+1-Y_{j} & \mathrm{when}\;j\mathrm{th}\;\mathrm{generated}\;\mathrm{packet}\;\mathrm{arrives}\;\mathrm{to}\;d. \end{array}\right.$$

where $Y_{j}$ is the interarrival time between the (j−1)th and *j*th arriving packet.

Notice that these (j−1)th and *j*th packets may be generated discontinuously, since between them, some updating packets may be discarded when they arrive and find the system full. Actually, this is exactly the difference between the finite and infinite status updating systems. Based on this observation, in [59], we have determined the average AoI and its stationary distribution for an infinite size status updating system with Bernoulli packet arrivals and geometric service time.

Denote the stationary AoI for the system with probabilistic packet preemption as $\Delta_{Ber/Geo/1/2^{*}/\eta }$. We define the time average AoI as follows, which is equal to the mean of the AoI because the age process is assumed to be ergodic. We have

(13) $$\begin{array}{cc} {\bar{\Delta }}_{Ber/Geo/1/2^{*}/\eta } & =\lim_{T\to \infty }\frac{1}{T}\sum_{k=1}^{T}a\left(k\right) \end{array}$$

(14) $$\begin{array}{cc} \; & =\lim_{T\to \infty }\frac{1}{T}\sum_{i=1}^{M_{T}}i\cdot \left|\left\{1\leq k\leq T:a(k)=i\right\}\right| \end{array}$$

(15) $$\begin{array}{cc} \; & =\sum_{i=1}^{\infty }i\cdot \pi_{i} \end{array}$$

where $\left|\left\{1\leq k\leq T:a(k)=i\right\}\right|$ is the times that the AoI takes value *i*, and $M_{T}=\max_{1\leq k\leq T}$a(k) is the maximal discrete AoI in *T* time slots. For each i≥1,

(16) $$\pi_{i}=\lim_{T\to \infty }\frac{\left|\left\{1\leq k\leq T:a(k)=i\right\}\right|}{T}$$

is the probability that the stationary AoI takes value *i*. In fact, the probability distribution $\left\{\pi_{i},i\geq 1\right\}$ forms the stationary distribution of the AoI $\Delta_{Ber/Geo/1/2^{*}/\eta }$.

The randomness of both packet arrivals and the service time in the server, along with the probabilistic packet preemption in the system’s buffer, together make the AoI at the destination change randomly. After one time slot, the value of AoI may increase by 1 if no packet is obtained or drop to the age of the obtained packet at that time if one such packet is successfully received. In order to fully describe these random dynamics of AoI, we propose to use a three-dimensional state vector to simultaneously record the changes of the AoI, the age of a packet in the server, and the age of the packet waiting in the buffer, and then constitute the three-dimensional stochastic process. Next, the steady-state of this multiple-dimensional age process is analyzed. To obtain the mean and the distribution of AoI, we define the PGF corresponding to the stationary distribution of the three-dimensional age process, from which both the AoI’s mean and its distribution can be obtained. The detailed analysis of the system’s AoI is given in Section 3.

## 3. AoI Analysis for Status Updating System with Probabilistic Packet Preemption

Define the three-dimensional state vector (n,m,l), where we use *n* to denote the AoI at destination *d*, and the other two parameters *m* and *l* are the ages of the packets in the system’s server and the buffer. In the *k*th time slot, if the server is busy while the buffer is empty, then $n_{k}$ and $m_{k}$ are greater than 0 but $l_{k}=0$. When both the server and the buffer are empty, we have $m_{k}=l_{k}=0$. In this case, the entire system is empty.

Consider the following three-dimensional age process

(17) $$AoI_{PP}=\left\{\left(n_{k},m_{k},l_{k}\right):n_{k}>m_{k}\geq l_{k}\geq 0,k\in N\right\}$$

where the subscript “PP” in expression (17) is the abbreviation of probabilistic preemption. Notice that when the system is empty, the last two parameters $m_{k}$ and $l_{k}$ are both equal to 0. When there are two packets in the system, i.e., one is in the server and the other is in the buffer, we show that the state components satisfy $n_{k}>m_{k}>l_{k}\geq 1$, since in a path from the source to the receiver, the packet ahead always has a greater age. It is clearly shown later that this relationship facilitates the derivation of probability generation function $H_{PP}\left(x\right)$, which is defined in Equation (20).

Define three random variables *A*, *B*, and *F* to represent whether a packet is generated in a time slot, if the service of the packet is completed, and if the arriving packet replaces the original one in the buffer. For each possible initial state vector, according to different realizations of r.v.s (A,B,F), the state transfers of the three-dimensional state vector (n,m,l) can be described specifically. We list all of them using Table 2. For example, the third row of the table shows that a packet of age *l* is in the buffer and a new packet arrives, since the r.v. *A* takes value 1. However, F=0 means that this new packet will not substitute the original one; meanwhile, B=0 implies that the packet service is not over at this time slot. Summarizing all these events, the beginning state vector (n,m,l) will transfer to (n+1,m+1,l+1) at the next time slot, and the transition probability is determined as p(1−γ)(1−η). The other cases in the third column of Table 2 are obtained through similar discussions.

From the state transfers given in Table 2 and the corresponding transition probabilities, we can establish all the stationary equations that characterize the steady-state of age process $AoI_{PP}$. Let $\pi_{\left(n,m,l\right)}$, n>m≥l≥0 be the probability that the process stays at the state vector (n,m,l) when it reaches the steady-state; we show that these stationary probabilities $\pi_{\left(n,m,l\right)}$ satisfy the following equations.

(18) $$\left\{\begin{array}{cc} \pi_{\left(n,m,l\right)}=\pi_{\left(n-1,m-1,l-1\right)}\left[\left(1-p\right)\left(1-\gamma \right)+p\left(1-\gamma \right)\left(1-\eta \right)\right] & \;(n>m>l\geq 2)\\ \pi_{\left(n,m,1\right)}=\pi_{\left(n-1,m-1,0\right)}p\left(1-\gamma \right)+\sum_{j=1}^{m-2}\pi_{\left(n-1,m-1,j\right)}p\left(1-\gamma \right)\eta & \;(n>m\geq 3)\\ \pi_{\left(n,2,1\right)}=\pi_{\left(n-1,1,0\right)}p\left(1-\gamma \right) & \;(n\geq 3)\\ \pi_{\left(n,m,0\right)}=\pi_{\left(n-1,m-1,0\right)}\left(1-p\right)\left(1-\gamma \right)\\ \;\;\;+\sum_{k=n}^{\infty }\pi_{\left(k,n-1,m-1\right)}\left[\left(1-p\right)\gamma +p\gamma \left(1-\eta \right)\right] & \;(n>m\geq 2)\\ \pi_{\left(n,1,0\right)}=\pi_{\left(n-1,0,0\right)}p\left(1-\gamma \right)\\ \;\;\;+\sum_{k=n}^{\infty }\pi_{\left(k,n-1,0\right)}p\gamma +\sum_{k=n}^{\infty }\sum_{j=1}^{n-2}\pi_{\left(k,n-1,j\right)}p\gamma \eta & \;(n\geq 3)\\ \pi_{\left(2,1,0\right)}=\pi_{\left(1,0,0\right)}p\left(1-\gamma \right)+\sum_{k=2}^{\infty }\pi_{\left(k,1,0\right)}p\gamma \\ \pi_{\left(n,0,0\right)}=\pi_{\left(n-1,0,0\right)}\left(1-p\right)+\sum_{k=n}^{\infty }\pi_{\left(k,n-1,0\right)}\left(1-p\right)\gamma & \;(n\geq 2)\\ \pi_{\left(1,0,0\right)}=\sum_{n=1}^{\infty }\pi_{\left(n,0,0\right)}p\gamma \end{array}\right.$$

We explain the stationary equations only for a part of the state vectors and show that the other equations in (18) can be determined in a similar manner. Firstly, for the fifth row of (18), the state vector (n,1,0) can be obtained from (n−1,0,0) assuming that a new packet arrives and enters the server directly, but the service does not end in a single time slot. Next, from the current state vector (k,n−1,0), k≥n, if the service of the age (n−1) packet is completed and a new packet arrives at the same time slot, it is observed that the packet of age (n−1) will be sent to the receiver, which makes the AoI change to *n* at next time slot. The new packet enters the server; thus, the middle parameter of the state vector changes to 1. This gives the expected state (n,1,0). Since in this case, the buffer is empty, when a new packet comes, it occupies the buffer directly, and no packet preemption occurs. At last, we consider the situation where the age process begins with an arbitrary state (k,n−1,j) where k>n−1>j≥1. As long as the packet service is completed and at the same time a new packet arrives preempting the original one in the buffer, again, we will obtain the state vector (n,1,0) after one time slot. Combining all of the above cases, the stationary equation corresponding to (n,1,0) is finally determined. In addition to the fifth row, we also explain the last equation in (18). Observing that in order to obtain the state vector (1,0,0), the receiver needs a packet of age 1, the system has then to be emptied. This state can be transferred to only from (n,0,0), and the service time of the newly arrived packet is restricted to be only one time slot.

To derive the expression of the average AoI ${\bar{\Delta }}_{Ber/Geo/1/2^{*}/\eta }$, we do not solve Equation (18) although this approach is feasible for the AoI analysis of tje current system. In our work [60], we analyzed the AoI of a status updating system with Ber/Geo/1/1, Ber/Geo/1/2, and $Ber/Geo/1/2^{*}$ queues, and the expression of the AoI’s stationary distribution was determined for each case. There, we completely solved the stationary equations for each system and obtained the explicit expression for every stationary probability. Notice that this work can be regarded as a discrete correspondence of the packet management of continuous AoI in [8,9]. Assuming all the probabilities $\pi_{\left(n,m,l\right)}$ have been determined by solving Equation (18), we have

(19) $$\begin{array}{c} \mathrm{Pr}\left\{\Delta_{Ber/Geo/1/2^{*}/\eta }=n\right\}=\left\{\begin{array}{cc} \pi_{\left(1,0,0\right)} & \;(n=1)\\ \pi_{\left(n,0,0\right)}+\sum_{l=0}^{n-2}\sum_{m=l+1}^{n-1}\pi_{\left(n,m,l\right)} & \;(n\geq 2) \end{array}\right. \end{array}$$

since the probability that the AoI takes each *n* is equal to the sum of all the stationary probabilities with the identical first component. Equation (19) gives the stationary distribution of the AoI, from which we can calculate the average value of AoI as

$${\bar{\Delta }}_{Ber/Geo/1/2^{*}/\eta }=\sum_{n=1}^{\infty }n\cdot \mathrm{Pr}\left\{\Delta_{Ber/Geo/1/2^{*}/\eta }=n\right\}$$

However, the number of calculations to solve Equation (18) may be large, and apart from this, extra computations are required to determine the AoI’s distribution according to Formula (19). Since the AoI is denoted by the first component, to obtain the distribution of AoI, we need to sum all the other state components. Notice that when the dimension of defined state vector is bigger, more calculations are required to determine the AoI’s distribution. Therefore, we must determine the mean of AoI and its distribution in another way, i.e., the probability generation function (PGF) method.

For 0<x≤1, define the probability generation function

(20) $$H_{PP}\left(x\right)=\sum_{n=1}^{\infty }x^{n}\mathrm{Pr}\left\{\Delta_{Ber/Geo/1/2^{*}/\eta }=n\right\}$$

and we write $H_{PP}\left(x\right)$ further as

$$\begin{array}{cc} H_{PP}\left(x\right) & =x\mathrm{Pr}\left\{\Delta_{Ber/Geo/1/2^{*}/\eta }=1\right\}+\sum_{n=2}^{\infty }x^{n}\mathrm{Pr}\left\{\Delta_{Ber/Geo/1/2^{*}/\eta }=n\right\} \end{array}$$

(21) $$\begin{array}{cc} \; & =x\pi_{\left(1,0,0\right)}+\sum_{n=2}^{\infty }x^{n}\left\{\pi_{\left(n,0,0\right)}+\sum_{l=0}^{n-2}\sum_{m=l+1}^{n-1}\pi_{\left(n,m,l\right)}\right\} \end{array}$$

(22) $$\begin{array}{cc} \; & =\sum_{n=1}^{\infty }x^{n}\pi_{\left(n,0,0\right)}+\sum_{n=2}^{\infty }x^{n}\sum_{l=0}^{n-2}\sum_{m=l+1}^{n-1}\pi_{\left(n,m,l\right)} \end{array}$$

(23) $$\begin{array}{cc} \; & =\sum_{n=1}^{\infty }x^{n}\pi_{\left(n,0,0\right)}+\sum_{l=0}^{\infty }\sum_{m=l+1}^{\infty }\sum_{n=m+1}^{\infty }x^{n}\pi_{\left(n,m,l\right)} \end{array}$$

(24) $$\begin{array}{cc} \; & =\sum_{n=1}^{\infty }x^{n}\pi_{\left(n,0,0\right)}+\sum_{m=1}^{\infty }\sum_{n=m+1}^{\infty }x^{n}\pi_{\left(n,m,0\right)}+\sum_{l=1}^{\infty }\sum_{m=l+1}^{\infty }\sum_{n=m+1}^{\infty }x^{n}\pi_{\left(n,m,l\right)} \end{array}$$

where in (21) we have used the probability expressions (19). Equation (23) is obtained by exchanging the summation order in (22). In Equation (24), we divide the PGF $H_{PP}\left(x\right)$ into three parts. It can be seen in the following paragraphs that the entire function (20) is obtained by determining these parts separately.

According to expression (20), immediately, we have

(25) $$H_{PP}\left(1\right)=1,\;{\left.\frac{dH_{PP}\left(x\right)}{dx}\right|}_{x=1}={\bar{\Delta }}_{Ber/Geo/1/2^{*}/\eta }$$

That is, the average AoI can be obtained from the PGF’s derivative at point x=1, and the probability that the steady state AoI equals *n* is determined by the coefficient before the term $x^{n}$ for every n≥1.

Now, we determine the PGF $H_{PP}\left(x\right)$. For 0<x≤1, define the functions

$$\begin{array}{cc} h_{1}\left(x\right) & =\sum_{n=1}^{\infty }x^{n}\pi_{\left(n,0,0\right)}\\ h_{2}\left(x\right) & =\sum_{m=1}^{\infty }\sum_{n=m+1}^{\infty }x^{n}\pi_{\left(n,m,0\right)}\\ h_{3}\left(x\right) & =\sum_{l=1}^{\infty }\sum_{m=l+1}^{\infty }\sum_{n=m+1}^{\infty }x^{n}\pi_{\left(n,m,l\right)} \end{array}$$

and

$$h_{2}^{\left(m\right)}\left(x\right)=\sum_{m=1}^{\infty }\sum_{n=m+1}^{\infty }x^{m}\pi_{\left(n,m,0\right)}$$

We first give the following lemma, from which the PGF $H_{PP}\left(x\right)$ can be determined completely.

**Lemma** **1.** *For the functions $h_{i}\left(x\right)$, 1≤i≤3 and $h_{2}^{\left(m\right)}\left(x\right)$, we have*

(26) $$h_{1}\left(x\right)=\frac{p\gamma M_{1}x}{1-(1-p)x}+\frac{(1-p)\gamma x}{1-(1-p)x}h_{2}^{\left(m\right)}\left(x\right)$$

(27) $$\begin{array}{c} h_{2}\left(x\right)=\frac{p(1-\gamma )x}{1-(1-p)(1-\gamma )x}h_{1}\left(x\right)+\frac{p\gamma x}{\left[1-(1-p)(1-\gamma )x][1-(1-\gamma )x\right]}h_{2}^{\left(m\right)}\left(x\right) \end{array}$$

(28) $$h_{3}\left(x\right)=\frac{p(1-\gamma )x}{1-(1-\gamma )x}h_{2}\left(x\right)$$

*and it is determined that*

(29) $$\begin{array}{c} h_{2}^{\left(m\right)}\left(x\right)=\frac{\left[\gamma +p^{2}\left(1-\gamma \right)\eta -\left(1-p\right)\left(1-\gamma \right)\left(1-p\eta \right)\gamma x\right]M_{2}x}{\left[1-(1-p)(1-\gamma )x][1-(1-\gamma )(1-p\eta )x\right]} \end{array}$$

*in which the numbers $M_{1}$ and $M_{2}$ are given as*

(30) $$\begin{array}{cc} M_{1} & =\frac{\left(1-p\right)\gamma^{2}}{\left(p+\gamma -2p\gamma \right)\gamma +p^{2}{\left(1-\gamma \right)}^{2}} \end{array}$$

(31) $$\begin{array}{cc} M_{2} & =\frac{p\gamma (1-\gamma )}{\left(p+\gamma -2p\gamma \right)\gamma +p^{2}{\left(1-\gamma \right)}^{2}} \end{array}$$

**Proof.**  Lemma 1 is proved in Appendix A. □

Using Lemma 1, we calculate the PGF $H_{PP}\left(x\right)$ as follows. Equation (24) shows

(32) $$\begin{array}{cc} H_{PP}\left(x\right) & =h_{1}\left(x\right)+h_{2}\left(x\right)+h_{3}\left(x\right)\\ \; & =h_{1}\left(x\right)+h_{2}\left(x\right)+\frac{p(1-\gamma )x}{1-(1-\gamma )x}h_{2}\left(x\right)\\ \; & =h_{1}\left(x\right)+\frac{1-(1-p)(1-\gamma )x}{1-(1-\gamma )x}(\frac{p(1-\gamma )x}{1-(1-p)(1-\gamma )x}h_{1}\left(x\right)\\ \; & \;\;+\frac{p\gamma x}{\left[1-(1-p)(1-\gamma )x][1-(1-\gamma )x\right]}h_{2}^{\left(m\right)}\left(x\right))\\ \; & =\frac{1-(1-p)(1-\gamma )x}{1-(1-\gamma )x}h_{1}\left(x\right)+\frac{p\gamma x}{{\left[1-\left(1-\gamma \right)x\right]}^{2}}h_{2}^{\left(m\right)}\left(x\right) \end{array}$$

where in (32) we have substituted Equation (27).

Using Equation (26) and merging the same terms, eventually, we obtain

(33) $$\begin{array}{cc} H_{PP}\left(x\right) & =\frac{p\gamma M_{1}x\left[1-\left(1-p\right)\left(1-\gamma \right)x\right]}{\left[1-(1-p)x][1-(1-\gamma )x\right]}\\ \; & \;+\frac{\gamma x\left\{1-\left(1-p\right)\left[2\left(1-\gamma \right)+p\gamma \right]x+{\left(1-p\right)}^{2}{\left(1-\gamma \right)}^{2}x^{2}\right\}}{\left[1-\left(1-p\right)x\right]{\left[1-\left(1-\gamma \right)x\right]}^{2}}h_{2}^{\left(m\right)}\left(x\right) \end{array}$$

in which the function $h_{2}^{\left(m\right)}\left(x\right)$ is given in Equation (29).

According to Formula (25), the average AoI of the system with probabilistic packet preemption is calculated in Theorem 1.

**Theorem** **1.** For the discrete time state updating system with a $Ber/Geo/1/2^{*}/\eta$ queue, assuming the packet waiting in the buffer can be preempted by following fresher packets with probability η, then the average age of information of this system is determined as

(34) $$\begin{array}{cc} \; & {\bar{\Delta }}_{Ber/Geo/1/2^{*}/\eta }=\frac{(p+\gamma -p\gamma )(p+\gamma )-p\gamma }{p\gamma }M_{1}+\frac{{\left(p+\gamma -p\gamma \right)}^{2}-p\gamma \left(1-p\right)}{p\gamma }\cdot {\left.\frac{dh_{2}^{\left(m\right)}\left(x\right)}{dx}\right|}_{x=1}\\ \; & \;\;\;+\frac{\left\{(p+\gamma -p\gamma )[1-3(1-p)(1-\gamma )]-2p\gamma (1-p)\right\}p\gamma +{\mathrm{Poly}}_{1}}{p^{2}\gamma^{2}}M_{2} \end{array}$$

*in which we define*

(35) $${\mathrm{Poly}}_{1}=\left[{\left(p+\gamma -p\gamma \right)}^{2}-p\gamma \left(1-p\right)\right]\left[2p\left(1-\gamma \right)+\left(1-p\right)\gamma \right]$$

*and the derivative of $h_{2}^{\left(m\right)}\left(x\right)$ at point 1 is calculated as*

(36) $${\left.\frac{dh_{2}^{\left(m\right)}\left(x\right)}{dx}\right|}_{x=1}=\left(\frac{1}{p+\gamma -p\gamma }+\frac{p(1-\gamma )(1-p\eta )}{\left(p+\gamma -p\gamma )[\gamma +p(1-\gamma )\eta \right]}\right)M_{2}$$

*Let $p=\rho_{d}\cdot \gamma$ and substitute numbers $M_{1}$, $M_{2}$; the average AoI is also written as*

(37) $$\begin{array}{cc} {\bar{\Delta }}_{Ber/Geo/1/2^{*}/\eta }=\; & \frac{1+\rho_{d}\left(1-2\gamma \right)+\rho_{d}^{2}{\left(1-\gamma \right)}^{2}+2\rho_{d}^{3}{\left(1-\gamma \right)}^{2}}{\rho_{d}\gamma \left[1+\rho_{d}\left(1-2\gamma \right)+\rho_{d}^{2}{\left(1-\gamma \right)}^{2}\right]}\\ \; & +\frac{\left(1-\gamma \right)+\rho_{d}\left(1-\gamma \right)\left(1-2\gamma \right)+\rho_{d}^{2}\left(1-\gamma \right)\left(1-\gamma +\gamma^{2}\right)}{1+\rho_{d}\left(1-2\gamma \right)+\rho_{d}^{2}{\left(1-\gamma \right)}^{2}}\{\frac{\left(2-\gamma \right)-\rho_{d}\gamma \left(1-\gamma \right)}{\gamma [1+\rho_{d}\left(1-\gamma \right)]}\\ & -\frac{\left(1-\gamma \right)-\rho_{d}\left(1-\gamma \right)\left[1+\gamma -\left(1-\gamma \right)\eta \right]+\rho_{d}^{2}\gamma^{2}\left(1-\gamma \right)\eta }{\gamma [1+\rho_{d}\left(1-\gamma \right)\left(1+\eta \right)+\rho_{d}^{2}{\left(1-\gamma \right)}^{2}\eta ]}\} \end{array}$$

*where $\rho_{d}=p/\gamma$ is defined as the discrete traffic load.*

**Proof.**  The average AoI is determined by first computing the derivative of $H_{PP}\left(x\right)$ in (33) and then letting x=1. Replacing parameter *p* with $\rho_{d}\cdot \gamma$, expression (37) is obtained eventually. Although a certain amount of calculation is required, all the computations are straight-forward.

Here, we only provide the details from obtaining Equation (36). From (29), we have

(38) $$\begin{array}{cc} {\left.\frac{dh_{2}^{\left(m\right)}\left(x\right)}{dx}\right|}_{x=1}= & {\left.\frac{d}{dx}\frac{\left[\gamma +p^{2}\left(1-\gamma \right)\eta -\left(1-p\right)\left(1-\gamma \right)\left(1-p\eta \right)\gamma x\right]M_{2}x}{\left[1-(1-p)(1-\gamma )x][1-(1-\gamma )(1-p\eta )x\right]}\right|}_{x=1}\\ = & {\left.\frac{d}{dx}\left(M_{2}\frac{\left[\gamma +p^{2}\left(1-\gamma \right)\eta \right]x-\left(1-p\right)\left(1-\gamma \right)\left(1-p\eta \right)\gamma x^{2}}{1-\left[\left(1-p\right)\left(1-\gamma \right)+\left(1-\gamma \right)\left(1-p\eta \right)\right]x+\left(1-p\right){\left(1-\gamma \right)}^{2}\left(1-p\eta \right)x^{2}}\right)\right|}_{x=1}\\ = & M_{2}\frac{{\mathrm{Poly}}_{2}\cdot \left(p+\gamma -p\gamma \right)\left[\gamma +p\left(1-\gamma \right)\eta \right]+\left[\gamma +p^{2}\left(1-\gamma \right)\eta -\left(1-p\right)\left(1-\gamma \right)\left(1-p\eta \right)\gamma \right]\cdot {\mathrm{Poly}}_{3}}{{\left(p+\gamma -p\gamma \right)}^{2}{\left[\gamma +p\left(1-\gamma \right)\eta \right]}^{2}} \end{array}$$

where

$${\mathrm{Poly}}_{2}=\gamma +p^{2}\left(1-\gamma \right)\eta -2\left(1-p\right)\left(1-\gamma \right)\left(1-p\eta \right)\gamma$$

and

$$\begin{array}{cc} {\mathrm{Poly}}_{3} & =\left(1-p\right)\left(1-\gamma \right)+\left(1-\gamma \right)\left(1-p\eta \right)-2\left(1-p\right){\left(1-\gamma \right)}^{2}\left(1-p\eta \right)\\ \; & =(1-p)(1-\gamma )[\gamma +p(1-\gamma )\eta ]+(p+\gamma -p\gamma )(1-\gamma )(1-p\eta ) \end{array}$$

Notice that

(39) $$\begin{array}{cc} \; & \gamma +p^{2}\left(1-\gamma \right)\eta -\left(1-p\right)\left(1-\gamma \right)\left(1-p\eta \right)\gamma \\ = & \gamma +p(1-\gamma )\eta -p(1-p)(1-\gamma )\eta -(1-p)(1-\gamma )(1-p\eta )\gamma \\ = & \gamma +p(1-\gamma )\eta -(1-p)(1-\gamma )[\gamma +p(1-\gamma )\eta ]\\ = & \left(p+\gamma -p\gamma )[\gamma +p(1-\gamma )\eta \right] \end{array}$$

Substituting (39) into (38) results in

(40) $$\begin{array}{cc} {\left.\frac{dh_{2}^{\left(m\right)}\left(x\right)}{dx}\right|}_{x=1} & =M_{2}\left(1-\frac{(1-p)(1-\gamma )(1-p\eta )\gamma }{\left(p+\gamma -p\gamma )[\gamma +p(1-\gamma )\eta \right]}+\frac{\left(1-p)(1-\gamma \right)}{p+\gamma -p\gamma }+\frac{\left(1-\gamma )(1-p\eta \right)}{\gamma +p(1-\gamma )\eta }\right)\\ \; & =M_{2}\left(\frac{1}{p+\gamma -p\gamma }+\frac{p(1-\gamma )(1-p\eta )}{\left(p+\gamma -p\gamma )[\gamma +p(1-\gamma )\eta \right]}\right) \end{array}$$

which is exactly Equation (36). □

Notice that in definition (20), for each n≥1, the coefficient of $x^{n}$ is the probability that the AoI equals *n*. In order to obtain these coefficients, we decompose the PGF $H_{PP}\left(x\right)$ into power series. This shows that

(41) $$\begin{array}{cc} H_{PP}\left(x\right) & =\frac{p\gamma M_{1}x}{\gamma -p}\left(\frac{(1-p)\gamma }{1-(1-p)x}-\frac{p(1-\gamma )}{1-(1-\gamma )x}\right)\\ \; & \;-\left(\frac{{\left(1-p\right)}^{2}\gamma^{2}M_{2}x^{2}}{\left(\gamma -p)[1-(1-p)x\right]}-\frac{p\gamma \left(1-p\right)\left(1-\gamma \right)M_{2}x^{2}}{\left(\gamma -p)[1-(1-\gamma )x\right]}+\frac{p\gamma M_{2}x^{2}}{{\left[1-(1-\gamma )x\right]}^{2}}\right)\\ \; & \;\times \left(\frac{\eta (1-p)(p+\gamma -p\gamma )}{\left(1-\eta )[1-(1-p)(1-\gamma )x\right]}-\frac{\left(1-p\eta )[\gamma +p(1-\gamma )\eta \right]}{\left(1-\eta )[1-(1-\gamma )(1-p\eta )x\right]}\right) \end{array}$$

when the preemption probability η≠1, while for the case η=1, we have

(42) $$\begin{array}{cc} H_{PP}\left(x\right) & =\frac{p\gamma M_{1}x}{\gamma -p}\left(\frac{(1-p)\gamma }{1-(1-p)x}-\frac{p(1-\gamma )}{1-(1-\gamma )x}\right)\\ \; & \;+\left(\frac{{\left(1-p\right)}^{2}\gamma^{2}M_{2}x^{2}}{\left(\gamma -p)[1-(1-p)x\right]}-\frac{p\gamma \left(1-p\right)\left(1-\gamma \right)M_{2}x^{2}}{\left(\gamma -p)[1-(1-\gamma )x\right]}+\frac{p\gamma M_{2}x^{2}}{{\left[1-(1-\gamma )x\right]}^{2}}\right)\\ \; & \;\times \frac{\gamma +p^{2}\left(1-\gamma \right)-{\left(1-p\right)}^{2}\left(1-\gamma \right)\gamma x}{{\left[1-(1-p)(1-\gamma )x\right]}^{2}} \end{array}$$

The details of obtaining Equations (41) and (42) are given in Appendix B. In Section 4, along with the average value of the AoI, we determine the AoI’s stationary distribution for two extreme cases: η=0 and η=1.

## 4. Stationary Age of Information under Two Extreme Cases

In this section, we determine the average AoI of the status updating system without packet preemption by setting η=0, and when the preemption probability η is equal to 1, the mean of the AoI for the $Ber/Geo/1/2^{*}$ queue modeled system is also derived. In addition, using Equations (41) and (42), the stationary distributions of the discrete AoI for two cases are also obtained.

**Theorem** **2.** Assuming the packet arrivals form a Bernoulli process and the service time is geometrically distributed, the average AoIs of the discrete time status updating system with Ber/Geo/1/2 and $Ber/Geo/1/2^{*}$ queues are calculated as

(43) $$\begin{array}{c} {\bar{\Delta }}_{Ber/Geo/1/2}=\frac{1}{\gamma }\left(\left(1-\gamma \right)+\frac{1}{\rho_{d}}+\frac{2\rho_{d}^{2}\left(1-\gamma \right)\left(1-\gamma /2\right)}{1+\rho_{d}\left(1-2\gamma \right)+\rho_{d}^{2}{\left(1-\gamma \right)}^{2}}\right) \end{array}$$

*and*

(44) $$\begin{array}{c} {\bar{\Delta }}_{Ber/Geo/1/2^{*}}=\frac{1}{\gamma }\left(\left(1-\gamma \right)+\frac{1}{\rho_{d}}+\frac{\rho_{d}^{2}\left(1-\gamma \right)\left[1+3\rho_{d}\left(1-\gamma \right)+\rho_{d}^{2}\left(1-\gamma \right)\left(1-2\gamma \right)\right]}{\left[1+\rho_{d}\left(1-2\gamma \right)+\rho_{d}^{2}{\left(1-\gamma \right)}^{2}\right]{\left[1+\rho_{d}\left(1-\gamma \right)\right]}^{2}}\right) \end{array}$$

*For each n≥1, the distribution of the AoI $\Delta_{Ber/Geo/1/2}$ is given by*

(45) $$\begin{array}{cc} \mathrm{Pr}\left\{\Delta_{Ber/Geo/1/2}=n\right\} & =\frac{p\gamma^{2}\left(1-p\right)M_{1}}{{\left(\gamma -p\right)}^{2}}{\left(1-p\right)}^{n}-\frac{\left(\gamma -p^{2}\right)\left(1-p\right)\gamma^{2}M_{2}}{{\left(\gamma -p\right)}^{2}}{\left(1-\gamma \right)}^{n-1}\\ \; & -\frac{p\gamma^{2}\left(1-p\right)M_{2}}{\gamma -p}\left(n-1\right){\left(1-\gamma \right)}^{n-1}+\frac{p\gamma^{2}M_{2}}{2}n\left(n-1\right){\left(1-\gamma \right)}^{n-2} \end{array}$$

*while when the system has full packet preemption, we show that*

(46) $$\begin{array}{cc} \; & \mathrm{Pr}\left\{\Delta_{Ber/Geo/1/2^{*}}=n\right\}\\ = & \frac{p\gamma M_{1}}{\gamma -p}\left(\gamma {\left(1-p\right)}^{n}-p{\left(1-\gamma \right)}^{n}\right)\\ & +\frac{\left(1-p\right)\left[\gamma^{2}+p\left(1-\gamma \right)\left(p+\gamma \right)\right]M_{2}}{\gamma -p}\left({\left(1-p\right)}^{n-1}-{\left[\left(1-p\right)\left(1-\gamma \right)\right]}^{n-1}\right)\\ & -\frac{\left(1-p\right)\gamma \left[p+2\left(1-p\right)\gamma \right]M_{2}}{\gamma -p}\left({\left(1-\gamma \right)}^{n-1}-{\left[\left(1-p\right)\left(1-\gamma \right)\right]}^{n-1}\right)+p\gamma M_{2}(A{\left(1-\gamma \right)}^{n-2}\\ & +B\left(n-1\right){\left(1-\gamma \right)}^{n-2}+C{\left[\left(1-p\right)\left(1-\gamma \right)\right]}^{n-2}+D\left(n-1\right){\left[\left(1-p\right)\left(1-\gamma \right)\right]}^{n-2}) \end{array}$$

*in which the coefficients A, B, C, and D are determined by*

(47) $$\begin{array}{cc} A & =\frac{2-p}{p^{3}}\left({\left(1-p\right)}^{2}\gamma -\frac{2\left(1-p\right)[\gamma +p^{2}\left(1-\gamma \right)]}{2-p}\right) \end{array}$$

(48) $$\begin{array}{cc} C & =-\frac{\left(1-p)(2-p\right)}{p^{3}}\left({\left(1-p\right)}^{2}\gamma -\frac{2\left(1-p\right)[\gamma +p^{2}\left(1-\gamma \right)]}{2-p}\right) \end{array}$$

(49) $$\begin{array}{cc} D & =-\frac{p^{2}\left(1-p\right)\cdot A+{\left(1-p\right)}^{2}\left[\gamma +p^{2}\left(1-\gamma \right)\right]}{p(2-p)} \end{array}$$

*and*

(50) $$B=[\gamma +p^{2}\left(1-\gamma \right)]-A-C-D$$

**Proof.**  We first derive two average AoIs in Equations (43) and (44) from the general expression (37). Let η be 0; then, no packet preemption will occur in the system’s buffer. The system’s queue model reduces to Ber/Geo/1/2, and from (37), we can obtain the average AoI ${\bar{\Delta }}_{Ber/Geo/1/2}$.

In this case, it is easy to show the last two terms within the brace of (37) can be calculated to be 1/γ. Thus, we have

$$\begin{array}{cc} \; & {\bar{\Delta }}_{Ber/Geo/1/2}={\left.{\bar{\Delta }}_{Ber/Geo/1/2^{*}/\eta }\right|}_{\eta =0}\\ = & \frac{1+\rho_{d}\left(1-2\gamma \right)+\rho_{d}^{2}{\left(1-\gamma \right)}^{2}+2\rho_{d}^{3}{\left(1-\gamma \right)}^{2}}{\rho_{d}\gamma \left[1+\rho_{d}\left(1-2\gamma \right)+\rho_{d}^{2}{\left(1-\gamma \right)}^{2}\right]}\\ & \;+\frac{\left(1-\gamma \right)+\rho_{d}\left(1-\gamma \right)\left(1-2\gamma \right)+\rho_{d}^{2}\left(1-\gamma \right)\left(1-\gamma +\gamma^{2}\right)}{\gamma [1+\rho_{d}\left(1-2\gamma \right)+\rho_{d}^{2}{\left(1-\gamma \right)}^{2}]}\\ = & \frac{1+\rho_{d}\left(2-3\gamma \right)+\rho_{d}^{2}\left(1-\gamma \right)\left(2-3\gamma \right)+\rho_{d}^{3}\left(1-\gamma \right)\left(3-3\gamma +\gamma^{2}\right)}{\rho_{d}\gamma \left[1+\rho_{d}\left(1-2\gamma \right)+\rho_{d}^{2}{\left(1-\gamma \right)}^{2}\right]} \end{array}$$

(51) $$\begin{array}{cc} = & \frac{1}{\rho_{d}\gamma }\left(1+\rho_{d}\left(1-\gamma \right)+\frac{2\rho_{d}^{3}\left(1-\gamma \right)\left(1-\gamma /2\right)}{1+\rho_{d}\left(1-2\gamma \right)+\rho_{d}^{2}{\left(1-\gamma \right)}^{2}}\right) \end{array}$$

(52) $$\begin{array}{cc} = & \frac{1}{\gamma }\left(\left(1-\gamma \right)+\frac{1}{\rho_{d}}+\frac{2\rho_{d}^{2}\left(1-\gamma \right)\left(1-\gamma /2\right)}{1+\rho_{d}\left(1-2\gamma \right)+\rho_{d}^{2}{\left(1-\gamma \right)}^{2}}\right) \end{array}$$

where in Equation (51) we use the method of long division.

For the other extreme case of η=1, obviously the general expression (37) gives the average AoI ${\bar{\Delta }}_{Ber/Geo/1/2^{*}}$. Similarly, we first determine the value of the last two terms within the brace. We show that the difference of the last two terms equals

(53) $$\frac{1}{\gamma }-\frac{\rho_{d}^{2}\left(1-\gamma \right)}{\gamma [1+\rho_{d}{\left(1-\gamma \right)}^{2}]}$$

thus, the average AoI ${\bar{\Delta }}_{Ber/Geo/1/2^{*}}$ is calculated as

$$\begin{array}{cc} \; & {\bar{\Delta }}_{Ber/Geo/1/2^{*}}={\left.{\bar{\Delta }}_{Ber/Geo/1/2^{*}/\eta }\right|}_{\eta =1} \end{array}$$

(54) $$\begin{array}{cc} = & {\bar{\Delta }}_{Ber/Geo/1/2}-\frac{\left(1-\gamma \right)+\rho_{d}\left(1-\gamma \right)\left(1-2\gamma \right)+\rho_{d}^{2}\left(1-\gamma \right)\left(1-\gamma +\gamma^{2}\right)}{1+\rho_{d}\left(1-2\gamma \right)+\rho_{d}^{2}{\left(1-\gamma \right)}^{2}}\cdot \frac{\rho_{d}^{2}\left(1-\gamma \right)}{\gamma {\left[1+\rho_{d}\left(1-\gamma \right)\right]}^{2}}\\ = & \frac{1}{\gamma }(\left(1-\gamma \right)+\frac{1}{\rho_{d}}+\frac{\rho_{d}^{2}\left(1-\gamma \right)\left(2-\gamma \right)}{1+\rho_{d}\left(1-2\gamma \right)+\rho_{d}^{2}{\left(1-\gamma \right)}^{2}} \end{array}$$

(55) $$\begin{array}{cc} & \;\;\;-\frac{\rho_{d}^{2}{\left(1-\gamma \right)}^{2}+\rho_{d}^{3}{\left(1-\gamma \right)}^{2}\left(1-2\gamma \right)+\rho_{d}^{4}{\left(1-\gamma \right)}^{2}\left(1-\gamma +\gamma^{2}\right)}{\left[1+\rho_{d}\left(1-2\gamma \right)+\rho_{d}^{2}{\left(1-\gamma \right)}^{2}\right]{\left[1+\rho_{d}\left(1-\gamma \right)\right]}^{2}}) \end{array}$$

(56) $$\begin{array}{cc} = & \frac{1}{\gamma }\left(\left(1-\gamma \right)+\frac{1}{\rho_{d}}+\frac{\rho_{d}^{2}\left(1-\gamma \right)\left[1+3\rho_{d}\left(1-\gamma \right)+\rho_{d}^{2}\left(1-\gamma \right)\left(1-2\gamma \right)\right]}{1+\rho_{d}\left(1-2\gamma \right)+\rho_{d}^{2}{\left(1-\gamma \right)}^{2}}\right) \end{array}$$

In Equation (54), since in the case of η=0, the difference of the last two terms is 1/γ, the average AoI ${\bar{\Delta }}_{Ber/Geo/1/2}$ is obtained. This equation also gives the exact gap between two average AoIs of the system with and without packet preemption. Notice that the latter term in (54) is always positive; then, the average AoI must become lower when the packet preemption strategy is applied. Equation (52) is substituted in (55), and in Equation (56), the expression of the average AoI ${\bar{\Delta }}_{Ber/Geo/1/2^{*}}$ is finally determined.

Next, the distribution of the discrete AoI is calculated. Before the expressions (45) and (46) are derived, we first verify that both (45) and (46) are proper probability distributions by providing a specific numerical example.

Numerical results of two AoI distributions. Let p=1/4 and γ=1/2.

Firstly, from Equations (30) and (31), two numbers $M_{1}$ and $M_{2}$ are determined to be

$$M_{1}=\frac{12}{17},\;M_{2}=\frac{4}{17}.$$

after some simple calculations, for each n≥1, the expression (45) gives

(57) $$\mathrm{Pr}\left\{\Delta_{Ber/Geo/1/2}=n\right\}=\frac{9}{17}{\left(\frac{3}{4}\right)}^{n}-\frac{21}{68}{\left(\frac{1}{2}\right)}^{n}-\frac{3}{68}\left(n-1\right){\left(\frac{1}{2}\right)}^{n-1}+\frac{1}{136}n\left(n-1\right){\left(\frac{1}{2}\right)}^{n-2}$$

To obtain the numerical result of Equation (46), it is necessary to determine the four coefficients *A*, *B*, *C*, and *D* according to expressions (47)–(50). We directly find that

$$A=-\frac{39}{2},\;C=\frac{117}{8},\;D=\frac{45}{32},\;B=4.$$

After some extra computations, it is shown that

(58) $$\begin{array}{cc} \mathrm{Pr}\left\{\Delta_{Ber/Geo/1/2^{*}}=n\right\} & =\frac{1}{2}{\left(\frac{3}{4}\right)}^{n}-\frac{105}{34}{\left(\frac{1}{2}\right)}^{n}\\ \; & \;+\frac{57}{17}{\left(\frac{3}{8}\right)}^{n}+\frac{2}{17}\left(n-1\right){\left(\frac{1}{2}\right)}^{n-2}+\frac{45}{1088}\left(n-1\right){\left(\frac{3}{8}\right)}^{n-2} \end{array}$$

It can be checked directly that the sum of both (57) and (58) from n=1 to *∞* are equal to 1. Therefore, expressions (45) and (46) indeed form the proper probability distributions.

In the following, by decomposing (41) and (42) further into several simplest rational fractions, we derive the explicit expressions of AoI distributions for the system with and without packet preemption.

First of all, for η=0, it is easy to prove that the last part of (41) is equal to

$$\frac{-\gamma }{1-(1-\gamma )x}$$

thus, we have following equations. This shows that

(59) $$\begin{array}{cc} \; & {\left.H_{PP}\left(x\right)\right|}_{\eta =0}\\ = & \frac{p\gamma M_{1}x}{\gamma -p}\left(\frac{(1-p)\gamma }{1-(1-p)x}-\frac{p(1-\gamma )}{1-(1-\gamma )x}\right)\\ & -\left(\frac{{\left(1-p\right)}^{2}\gamma^{2}M_{2}x^{2}}{\left(\gamma -p)[1-(1-p)x\right]}-\frac{p\gamma \left(1-p\right)\left(1-\gamma \right)M_{2}x^{2}}{\left(\gamma -p)[1-(1-\gamma )x\right]}+\frac{p\gamma M_{2}x^{2}}{{\left[1-(1-\gamma )x\right]}^{2}}\right)\cdot \frac{-\gamma }{1-(1-\gamma )x}\\ = & \frac{p\gamma^{2}\left(1-p\right)M_{1}x}{\left(\gamma -p)[1-(1-p)x\right]}-\frac{p^{2}\gamma \left(1-\gamma \right)M_{1}x}{\left(\gamma -p)[1-(1-\gamma )x\right]}\\ & +\frac{{\left(1-p\right)}^{2}\gamma^{3}M_{2}x^{2}}{\left(\gamma -p)[1-(1-p)x][1-(1-\gamma )x\right]}-\frac{p\gamma^{2}\left(1-p\right)\left(1-\gamma \right)M_{2}x^{2}}{\left(\gamma -p\right){\left[1-\left(1-\gamma \right)x\right]}^{2}}+\frac{p\gamma^{2}M_{2}x^{2}}{{\left[1-\left(1-\gamma \right)x\right]}^{3}}\\ = & \frac{p\gamma^{2}\left(1-p\right)M_{1}x}{\left(\gamma -p)[1-(1-p)x\right]}-\frac{p^{2}\gamma \left(1-\gamma \right)M_{1}x}{\left(\gamma -p)[1-(1-\gamma )x\right]}\\ & +\frac{{\left(1-p\right)}^{2}\gamma^{3}M_{2}x^{2}}{\left(\gamma -p\right)}\left(\frac{1-p}{\left(\gamma -p)[1-(1-p)x\right]}-\frac{1-\gamma }{\left(\gamma -p)[1-(1-\gamma )x\right]}\right)\\ & -\frac{p\gamma^{2}\left(1-p\right)\left(1-\gamma \right)M_{2}x^{2}}{\left(\gamma -p\right){\left[1-\left(1-\gamma \right)x\right]}^{2}}+\frac{p\gamma^{2}M_{2}x^{2}}{{\left[1-\left(1-\gamma \right)x\right]}^{3}}\\ = & \left(\frac{p\gamma^{2}\left(1-p\right)M_{1}x}{\gamma -p}+\frac{{\left(1-p\right)}^{3}\gamma^{3}M_{2}x^{2}}{{\left(\gamma -p\right)}^{2}}\right)\sum_{n=0}^{\infty }{\left[\left(1-p\right)x\right]}^{n}\\ & -\left(\frac{p^{2}\gamma \left(1-\gamma \right)M_{1}x}{\gamma -p}+\frac{{\left(1-p\right)}^{2}\left(1-\gamma \right)\gamma^{3}M_{2}x^{2}}{{\left(\gamma -p\right)}^{2}}\right)\sum_{n=0}^{\infty }{\left[\left(1-\gamma \right)x\right]}^{n}\\ & -\frac{p\gamma^{2}\left(1-p\right)\left(1-\gamma \right)M_{2}x^{2}}{\gamma -p}\sum_{n=1}^{\infty }n{\left[\left(1-\gamma \right)x\right]}^{n-1}+\frac{p\gamma^{2}M_{2}x^{2}}{2}\sum_{n=2}^{\infty }n\left(n-1\right){\left[\left(1-\gamma \right)x\right]}^{n-2} \end{array}$$

Taking the coefficient before $x^{n}$, we find that

(60) $$\begin{array}{cc} \; & \mathrm{Pr}\left\{\Delta_{Ber/Geo/1/2}=n\right\}\\ = & \left(\frac{p\gamma^{2}\left(1-p\right)M_{1}}{\gamma -p}{\left(1-p\right)}^{n-1}+\frac{{\left(1-p\right)}^{3}\gamma^{3}M_{2}}{{\left(\gamma -p\right)}^{2}}{\left(1-p\right)}^{n-2}\right)\\ & \;-\left(\frac{p^{2}\gamma \left(1-\gamma \right)M_{1}}{\gamma -p}{\left(1-\gamma \right)}^{n-1}+\frac{{\left(1-p\right)}^{2}\left(1-\gamma \right)\gamma^{3}M_{2}}{{\left(\gamma -p\right)}^{2}}{\left(1-\gamma \right)}^{n-2}\right)\\ & \;-\frac{p\gamma^{2}\left(1-p\right)\left(1-\gamma \right)M_{2}}{\gamma -p}\left(n-1\right){\left(1-\gamma \right)}^{n-2}+\frac{p\gamma^{2}M_{2}}{2}n\left(n-1\right){\left(1-\gamma \right)}^{n-2}\\ = & \frac{p\gamma^{2}\left(1-p\right)M_{1}}{{\left(\gamma -p\right)}^{2}}{\left(1-p\right)}^{n}-\frac{\left(\gamma -p^{2}\right)\left(1-p\right)\gamma^{2}M_{2}}{{\left(\gamma -p\right)}^{2}}{\left(1-\gamma \right)}^{n-1}\\ & \;-\frac{p\gamma^{2}\left(1-p\right)M_{2}}{\gamma -p}\left(n-1\right){\left(1-\gamma \right)}^{n-1}+\frac{p\gamma^{2}M_{2}}{2}n\left(n-1\right){\left(1-\gamma \right)}^{n-2} \end{array}$$

This gives the stationary distribution (45) for the system without packet preemption.

On the other hand, when η is equal to 1, the system has full packet preemption. Factoring the PGF in Equation (42), we can also determine the stationary distribution of the AoI $\Delta_{Ber/Geo/1/2^{*}}$ by taking the coefficients of terms $x^{n}$ for each n≥1. We give the explicit decomposition below, from which the distribution of AoI for the system with packet preemption is obtained explicitly.

From Equation (42), we show that

(61) $$\begin{array}{cc} \; & {\left.H_{PP}\left(x\right)\right|}_{\eta =1}\\ = & \frac{p\gamma M_{1}x}{\gamma -p}\left(\frac{(1-p)\gamma }{1-(1-p)x}-\frac{p(1-\gamma )}{1-(1-\gamma )x}\right)+\frac{{\left(1-p\right)}^{2}\gamma^{2}M_{2}x^{2}}{\gamma -p}(\frac{\gamma^{2}+p\left(1-\gamma \right)\left(p+\gamma \right)}{\gamma^{2}\left[1-\left(1-p\right)x\right]}\\ & -\frac{\left(1-\gamma \right)[\gamma^{2}+p\left(1-\gamma \right)\left(p+\gamma \right)]}{\gamma^{2}\left[1-\left(1-p\right)\left(1-\gamma \right)x\right]}-\frac{p(1-\gamma )(p+\gamma -p\gamma )}{\gamma {\left[1-\left(1-p\right)\left(1-\gamma \right)x\right]}^{2}})\\ & -\frac{p\gamma \left(1-p\right)\left(1-\gamma \right)M_{2}x^{2}}{\gamma -p}(\frac{p+2(1-p)\gamma }{p[1-(1-\gamma )x]}-\frac{\left(1-p)[p+2(1-p)\gamma \right]}{p[1-(1-p)(1-\gamma )x]}\\ & -\frac{\left(1-p)(p+\gamma -p\gamma \right)}{{\left[1-\left(1-p\right)\left(1-\gamma \right)x\right]}^{2}})+p\gamma M_{2}x^{2}(\frac{A}{1-(1-\gamma )x}+\frac{B}{{\left[1-\left(1-\gamma \right)x\right]}^{2}}\\ & +\frac{C}{1-(1-p)(1-\gamma )x}+\frac{D}{{\left[1-\left(1-p\right)\left(1-\gamma \right)x\right]}^{2}}) \end{array}$$

in which we determine

(62) $$\begin{array}{cc} A & =\frac{2-p}{p^{3}}\left({\left(1-p\right)}^{2}\gamma -\frac{2\left(1-p\right)[\gamma +p^{2}\left(1-\gamma \right)]}{2-p}\right) \end{array}$$

(63) $$\begin{array}{cc} C & =-\frac{\left(1-p)(2-p\right)}{p^{3}}\left({\left(1-p\right)}^{2}\gamma -\frac{2\left(1-p\right)[\gamma +p^{2}\left(1-\gamma \right)]}{2-p}\right) \end{array}$$

(64) $$\begin{array}{cc} D & =-\frac{p^{2}\left(1-p\right)\cdot A+{\left(1-p\right)}^{2}\left[\gamma +p^{2}\left(1-\gamma \right)\right]}{p(2-p)} \end{array}$$

and

(65) $$B=[\gamma +p^{2}\left(1-\gamma \right)]-A-C-D$$

Obtaining the second and the third row of (61) is not hard, while for the last row, we give some derivation details in Appendix C. Following the same procedures as those used to obtain (60), according to Equation (61), the probability that a stationary AoI equals each *n* is determined by the coefficient of the term $x^{n}$.

(66) $$\begin{array}{cc} \; & {\left.H_{PP}\left(x\right)\right|}_{\eta =1}\\ = & \frac{p\gamma M_{1}}{\gamma -p}\left(\left(1-p\right)\gamma {\left(1-p\right)}^{n-1}-p\left(1-\gamma \right){\left(1-\gamma \right)}^{n-1}\right)+\frac{{\left(1-p\right)}^{2}\gamma^{2}M_{2}}{\gamma -p}\\ & \times (\frac{\gamma^{2}+p\left(1-\gamma \right)\left(p+\gamma \right)}{\gamma^{2}}{\left(1-p\right)}^{n-2}-\frac{\left(1-\gamma \right)[\gamma^{2}+p\left(1-\gamma \right)\left(p+\gamma \right)]}{\gamma^{2}}{\left[\left(1-p\right)\left(1-\gamma \right)\right]}^{n-2}\\ & -\frac{p(1-\gamma )(p+\gamma -p\gamma )}{\gamma }\left(n-1\right){\left[\left(1-p\right)\left(1-\gamma \right)\right]}^{n-2})-\frac{p\gamma \left(1-p\right)\left(1-\gamma \right)M_{2}}{\gamma -p}\\ & \times (\frac{p+2(1-p)\gamma }{p}{\left(1-\gamma \right)}^{n-2}-\frac{\left(1-p)[p+2(1-p)\gamma \right]}{p}{\left[\left(1-p\right)\left(1-\gamma \right)\right]}^{n-2}\\ & -\left(1-p\right)\left(p+\gamma -p\gamma \right)\left(n-1\right){\left[\left(1-p\right)\left(1-\gamma \right)\right]}^{n-2})+p\gamma M_{2}(A{\left(1-\gamma \right)}^{n-2}\\ & +B\left(n-1\right){\left(1-\gamma \right)}^{n-2}+C{\left[\left(1-p\right)\left(1-\gamma \right)\right]}^{n-2}+D\left(n-1\right){\left[\left(1-p\right)\left(1-\gamma \right)\right]}^{n-2})\\ = & \frac{p\gamma M_{1}}{\gamma -p}\left(\gamma {\left(1-p\right)}^{n}-p{\left(1-\gamma \right)}^{n}\right)\\ & +\frac{\left(1-p\right)\left[\gamma^{2}+p\left(1-\gamma \right)\left(p+\gamma \right)\right]M_{2}}{\gamma -p}\left({\left(1-p\right)}^{n-1}-{\left[\left(1-p\right)\left(1-\gamma \right)\right]}^{n-1}\right)\\ & -\frac{\left(1-p\right)\gamma \left[p+2\left(1-p\right)\gamma \right]M_{2}}{\gamma -p}\left({\left(1-\gamma \right)}^{n-1}-{\left[\left(1-p\right)\left(1-\gamma \right)\right]}^{n-1}\right)+p\gamma M_{2}(A{\left(1-\gamma \right)}^{n-2}\\ & +B\left(n-1\right){\left(1-\gamma \right)}^{n-2}+C{\left[\left(1-p\right)\left(1-\gamma \right)\right]}^{n-2}+D\left(n-1\right){\left[\left(1-p\right)\left(1-\gamma \right)\right]}^{n-2}) \end{array}$$

which determines the distribution of AoI $\Delta_{Ber/Geo/1/2^{*}}$.

So far, in Equations (52), (56), (60) and (66), we have obtained all the results in Theorem 2; thus, the proof is completed. □

Actually, we have obtained the explicit expression of AoI’s distribution for the system with packet preemption in our early work [60]. Earlier in this paper, we explain that solving the stationary equations is feasible for the easy situations but cannot be generalized when the system structure or queue models become complex. In [60], we focused on the discrete time system with three queues, i.e., the Ber/Geo/1/1, Ber/Geo/1/2, and $Ber/Geo/1/2^{*}$, and named them “*discrete packet management strategies*”. There, we determined the AoI’s stationary distribution for each system, and all the cases are dealt with by solving the stationary equations directly. Although the calculations are long—even tedious—these methods still have great significance, especially when the general status updating system is considered where the packet arrival process or the packet service process is arbitrary. It is with these methods that the analysis of discrete AoI can break through the limitation of the memoryless property that is imposed on the packet arrival and packet service processes in the SHS approach.

In [9], based on graphical arguments of the age process, the authors determined the average continuous AoI for the system with M/M/1/2 and $M/M/1/2^{*}$ queues as

(67) $$\Delta_{M/M/1/2}=\frac{1}{\mu }\left(1+\frac{1}{\rho }+\frac{2\rho^{2}}{1+\rho +\rho^{2}}\right)$$

and

(68) $$\Delta_{M/M/1/2^{*}}=\frac{1}{\mu }\left(1+\frac{1}{\rho }+\frac{\rho^{2}\left(1+3\rho +\rho^{2}\right)}{\left(1+\rho +\rho^{2}\right){\left(1+\rho \right)}^{2}}\right)$$

In addition, in previous work [58], we have proved that the mean of the AoI for a bufferless discrete time status updating system is equal to

(69) $${\bar{\Delta }}_{Ber/Geo/1/1}=\frac{1}{\gamma }\left(\left(1-\gamma \right)+\frac{1}{\rho_{d}}+\frac{\rho_{d}}{1/\left(1-\gamma \right)+\rho_{d}}\right)$$

while the corresponding continuous system with an M/M/1/1 queue has the average AoI

(70) $${\bar{\Delta }}_{M/M/1/1}=\frac{1}{\mu }\left(1+\frac{1}{\rho }+\frac{\rho }{1+\rho }\right)$$

which was also given in [9].

We list Equations (43), (44) and (67)–(70) in Table 3—notice that this table has been given previously in Table 1 except for the last row, which gives the average continuous and discrete AoI for an infinite size status updating system. The mean of the discrete AoI $\Delta_{Ber/Geo/1/\infty }$ was obtained recently in our work [59]. It is observed that apart from some additional product factors, the expressions of discrete AoI means for the system with Bernoulli packet arrivals and geometric service times are identical to those of the continuous system’s average AoI, which uses the Poisson-exponential assumptions. So far, we have obtained enough evidence to propose the following relationship between the mean of discrete and continuous AoIs:

(71) $$\mu \cdot {\bar{\Delta }}_{M/M/1/c}={\left.\gamma \cdot {\bar{\Delta }}_{Ber/Geo/1/c}\right|}_{\gamma =0}\;\mathrm{then}\;\mathrm{replacing}\;\rho_{d}\;\mathrm{by}\;\rho$$

It is interesting and meaningful to verify the correspondence (71) by calculating the average AoI for more continuous time and discrete time systems. For example, determining the mean of AoI assuming the M/M/1/c queue is used in the continuous time system and for the discrete time systems with general Ber/Geo/1/c queues, where the system’s size *c* is larger than 2.

## 5. Numerical Simulation

**Figure 3:**
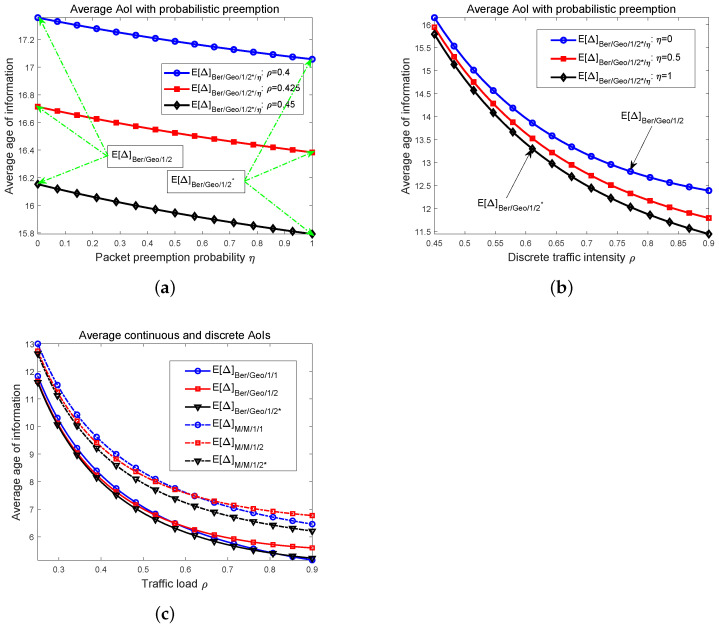
(**a**) Average AoI versus preemption probability η (different traffic load). (**b**) Average AoI versus traffic load $\rho_{d}$ (different preemption intensity). (**c**) Comparisons of average discrete AoI and average continuous AoI.

**Figure 4:**
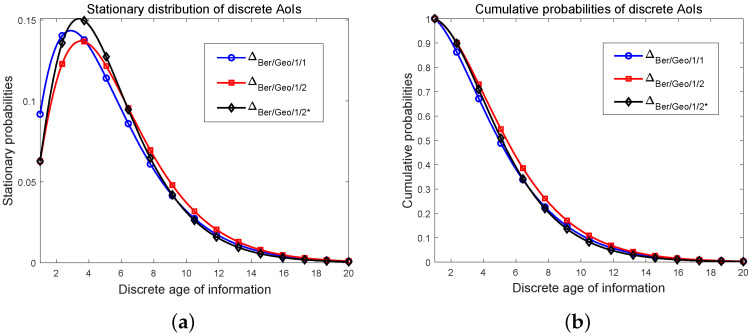
(**a**) Stationary distributions of discrete AoI for bufferless system and the system with and without packet preemption. (**b**) The cumulative probabilities of three discrete AoIs.

We provide the numerical results in this section. For general preemption probability, in the first two plots of Figure 3, we illustrate the relationships between the average AoI ${\bar{\Delta }}_{Ber/Geo/1/2^{*}/\eta }$ and the packet preemption probability η, and the traffic load $\rho_{d}$, respectively. The means of three discrete AoIs including ${\bar{\Delta }}_{Ber/Geo/1/1}$, ${\bar{\Delta }}_{Ber/Geo/1/2}$, and ${\bar{\Delta }}_{Ber/Geo/1/2^{*}}$ are plotted in Figure 3c. For comparison, we also provide the numerical simulations of corresponding average continuous AoIs. At last, for three discrete AoIs, we depict their distribution curves and the cumulative probabilities in Figure 4. Notice that in our work [58], the distribution of the AoI for the bufferless system was obtained as

(72) $$\mathrm{Pr}\left\{\Delta_{Ber/Geo/1/1}=n\right\}=\frac{p\left(1-p\right)\gamma^{3}\left[{\left(1-p\right)}^{n}-{\left(1-\gamma \right)}^{n}\right]}{\left(p+\gamma -p\gamma \right){\left(\gamma -p\right)}^{2}}-\frac{{\left(p\gamma \right)}^{2}n{\left(1-\gamma \right)}^{n}}{\left(p+\gamma -p\gamma )(\gamma -p\right)}$$

For three different traffic loads $\rho_{d}$, we first draw the graphs between the average AoI ${\bar{\Delta }}_{Ber/Geo/1/2^{*}/\eta }$ and the preemption probability η. It is understandable that replacing the packet in a buffer with a fresher one can decrease the average AoI at the destination, and the numerical results in Figure 3a show that this trend is consistent as the preemption probability becomes large; that is, the mean of the AoI is decreasing monotonically when η increases. We mark the values at two extreme points where η=0 and η=1, which gives the average AoI ${\bar{\Delta }}_{Ber/Geo/1/2}$ and ${\bar{\Delta }}_{Ber/Geo/1/2^{*}}$. Notice that the closer to η=0, the more similar the behavior of the system becomes to that of a system using Ber/Geo/1/2 queues, and when η gradually gets to 1, a status updating system with $Ber/Geo/1/2^{*}$ queue is finally obtained. The three curves in Figure 3a also show that as the traffic load $\rho_{d}$ increases from 0.4 to 0.45, the average AoI of the system with probabilistic packet preemption is reduced; thus, the timeliness performance is improved.

In Figure 3b, for three settings of preemption probabilities, i.e., η=0, η=0.5 and η=1, the relationships of the average AoI versus traffic intensity $\rho_{d}$ are illustrated. The topmost curve gives the average AoI of the system without packet preemption because for the case of η=0, the average AoI reduces to ${\bar{\Delta }}_{Ber/Geo/1/2}$. On the other hand, the curve at the bottom corresponds to the AoI’s mean of the system that has full packet preemption. In order to make the differences among these graphs more significant, we draw the results in the range $\rho_{d}\geq 0.45$. Three curves in Figure 3b clearly show that the timeliness of the system with complete packet preemption is the best, since when η is set to 1, the system’s average AoI is the lowest. Since the results in Figure 3a show that the average AoI is monotonically decreasing when η increases, the graphs of the AoI’s mean for a system with probabilistic packet preemption is located between the blue and the black lines in Figure 3b, such as the red line, which denotes the average AoI ${\bar{\Delta }}_{Ber/Geo/1/2^{*}/0.5}$. In addition, the gaps between these curves are not significant for small $\rho_{d}$s but become large as $\rho_{d}$ increases.

From $\rho_{d}=0.15$ to 0.9, we depict both the average discrete AoIs and the corresponding continuous average AoIs in Figure 3c for a bufferless system and size 2 status updating system with and without preemption. Continuous AoIs are denoted by dashed lines, and we use solid lines to represent the discrete AoIs. First of all, all the curves are decreasing as $\rho_{d}$ becomes large, and the gaps between them are gradually apparent. For three continuous AoIs, it is observed that the average AoI ${\bar{\Delta }}_{M/M/1/2^{*}}$ is the lowest in all the range of traffic load ρ. For the other two status updating systems, it is found that the system with an M/M/1/2 queue has a lower average AoI when ρ takes small values, while for high ρ, the average AoI of the bufferless system is smaller, and thus the timeliness is better. These results are the same for the graphs of discrete AoIs. Notice that when the discrete traffic intensity $\rho_{d}$ is extremely large (near 0.9), the numerical results show that the average AoI ${\bar{\Delta }}_{Ber/Geo/1/1}$ can be even smaller than ${\bar{\Delta }}_{Ber/Geo/1/2^{*}}$.

In Figure 3c, both continuous and discrete average AoIs are monotonically decreasing in the whole range of $\rho_{d}$; however, the monotonicity of the curve between the average AoI and $\rho_{d}$ can only be maintained for small-size status updating systems. It is known that the average AoI of an infinite size system, i.e., ${\bar{\Delta }}_{M/M/1/\infty }$, is not monotonic when the traffic load varies from 0 to 1. Thus, for a size *c* status updating system with Bernoulli packet arrivals and geometrically distributed service time, there must be a *critical size*$c^{*}$ such that when $c<c^{*}$, the mean of the system’s AoI ${\bar{\Delta }}_{Ber/Geo/1/c}$ is monotonically decreasing as $\rho_{d}$ tends to 1. In contrast, for those cases where the system size $c\geq c^{*}$, the curve has a valley, and an optimal $\rho_{d}$ exists at which the average AoI is minimized. Similarly, for the continuous average AoI ${\bar{\Delta }}_{M/M/1/c}$ of the system with general size *c*, a $c^{*}$ also exists so that ${\bar{\Delta }}_{M/M/1/c}$ is always decreasing when $c<c^{*}$ and the graph of ${\bar{\Delta }}_{M/M/1/c}$ first falls and then rises for those *c*s where $c\geq c^{*}$. In addition, from the alternation of ${\bar{\Delta }}_{M/M/1/1}$ and ${\bar{\Delta }}_{M/M/1/2}$, and also of ${\bar{\Delta }}_{Ber/Geo/1/1}$ and ${\bar{\Delta }}_{Ber/Geo/1/2}$, we can infer that, as a function of *c*, the graphs of ${\bar{\Delta }}_{M/M/1/c}$ and ${\bar{\Delta }}_{Ber/Geo/1/c}$ are not monotonic.

At last, the distribution curves and cumulative probabilities of three discrete AoIs are depicted in Figure 4, in which we set a relatively large $\rho_{d}$ to make the difference between them clear. On the whole, these curves are similar. In Figure 4a, from the distributions of AoI $\Delta_{Ber/Geo/1/1}$ to that of $\Delta_{Ber/Geo/1/2}$, the peak of the curve decreases and the point at which the peak stationary probability is achieved moves slightly to the right. As the AoI becomes large, the distribution curve of the system with the Ber/Geo/1/1 queue drops more sharply. The distribution corresponding to $\Delta_{Ber/Geo/1/2^{*}}$ has the largest peak value pf all of three discrete AoIs, and the descent speed is the fastest when the value of AoI is large. In addition, it seems that this maximal probability is taken at the same discrete AoI as that of the distribution of $\Delta_{Ber/Geo/1/2}$. We also provide the cumulative probabilities of three discrete AoIs in Figure 4b.

## 6. Conclusions

In this paper, we consider the stationary AoI of a size 2 status updating system where the packet waiting in the buffer can be preempted by fresher packets with the given probability η. We show that this phenomenon may occur in the energy-harvest (EH) nodes of wireless sensor networks where the charging process is stochastic. We constitute a three-dimensional age process and derive the general expression of the system’s average AoI using the PGF method. Let η=0 and η=1; the mean of two discrete AoIs ${\bar{\Delta }}_{Ber/Geo/1/2}$ and ${\bar{\Delta }}_{Ber/Geo/1/2}$ are determined, and the exact distribution expressions of both AoIs are also obtained by writing the PGF as the power series.

We propose the idea and methods for the analysis of discrete AoIs—that is, constituting multiple-dimensional age processes and applying the PGF method. A detailed introduction is given to exhibit the usage of the idea and methods to more discrete time status updating systems. With this paper, we have shown how the AoI of basic discrete system is characterized, while in further work, we will focus on the age analysis of systems with a more general structure, such as systems with multi-sources and systems with multi-hop packet transmission. As one part of the AoI theory, we believe that the research into discrete AoI deserves more attention.

## Figures and Tables

**Table 1:** Some formulas of the average continuous and average discrete age of information.

Average Continuous and Average Discrete AoIs
${\bar{\Delta }}_{M/M/1/1}=\frac{1}{\mu }\left(1+\frac{1}{\rho }+\frac{\rho }{1+\rho }\right)$
${\bar{\Delta }}_{Ber/Geo/1/1}=\frac{1}{\gamma }\left(\left(1-\gamma \right)+\frac{1}{\rho_{d}}+\frac{\rho_{d}}{1/\left(1-\gamma \right)+\rho_{d}}\right)$
${\bar{\Delta }}_{M/M/1/2}=\frac{1}{\mu }\left(1+\frac{1}{\rho }+\frac{2\rho^{2}}{1+\rho +\rho^{2}}\right)$
${\bar{\Delta }}_{Ber/Geo/1/2}=\frac{1}{\gamma }\left(\left(1-\gamma \right)+\frac{1}{\rho_{d}}+\frac{2\rho_{d}^{2}\left(1-\gamma \right)\left(1-\gamma /2\right)}{1+\rho_{d}\left(1-2\gamma \right)+\rho_{d}^{2}{\left(1-\gamma \right)}^{2}}\right)$
${\bar{\Delta }}_{M/M/1/2^{*}}=\frac{1}{\mu }\left(1+\frac{1}{\rho }+\frac{\rho^{2}\left(1+3\rho +\rho^{2}\right)}{\left(1+\rho +\rho^{2}\right){\left(1+\rho \right)}^{2}}\right)$
${\bar{\Delta }}_{Ber/Geo/1/2^{*}}=\frac{1}{\gamma }\left(\left(1-\gamma \right)+\frac{1}{\rho_{d}}+\frac{\rho_{d}^{2}\left(1-\gamma \right)\left[1+3\rho_{d}\left(1-\gamma \right)+\rho_{d}^{2}\left(1-\gamma \right)\left(1-2\gamma \right)\right]}{\left[1+\rho_{d}\left(1-2\gamma \right)+\rho_{d}^{2}{\left(1-\gamma \right)}^{2}\right]{\left[1+\rho_{d}\left(1-\gamma \right)\right]}^{2}}\right)$

**Table 2:** The state vector transfers of age process $AoI_{PP}$.

Initial State Vector	Considered r.v.s	Realizations and Next State Vector
	(A=0,B)	(0,0):(n+1,m+1,l+1)
		(0,1):(m+1,l+1,0)
$\begin{array}{c} (n,m,l),\\ n>m>l\geq 1 \end{array}$	(A=1,B,F)	(1,0,0):(n+1,m+1,l+1)
		(1,0,1):(n+1,m+1,1)
		(1,1,0):(m+1,l+1,0)
		(1,1,1):(m+1,1,0)
	(A,B)	(0,0):(n+1,m+1,0)
$\begin{array}{c} (n,m,0),\\ n>m\geq 1 \end{array}$		(0,1):(m+1,0,0)
		(1,0):(n+1,m+1,1)
		(1,1):(m+1,1,0)
	A=0	(n+1,0,0)
$\begin{array}{c} (n,0,0),\\ n\geq 1 \end{array}$	(A=1,B)	(1,0):(n+1,1,0)
		(1,1):(1,0,0)

**Table 3:** Some formulas of the average continuous and average discrete age of information.

Average Continuous and Average Discrete AoIs
${\bar{\Delta }}_{M/M/1/1}=\frac{1}{\mu }\left(1+\frac{1}{\rho }+\frac{\rho }{1+\rho }\right)$
${\bar{\Delta }}_{Ber/Geo/1/1}=\frac{1}{\gamma }\left(\left(1-\gamma \right)+\frac{1}{\rho_{d}}+\frac{\rho_{d}}{1/\left(1-\gamma \right)+\rho_{d}}\right)$
${\bar{\Delta }}_{M/M/1/2}=\frac{1}{\mu }\left(1+\frac{1}{\rho }+\frac{2\rho^{2}}{1+\rho +\rho^{2}}\right)$
${\bar{\Delta }}_{Ber/Geo/1/2}=\frac{1}{\gamma }\left(\left(1-\gamma \right)+\frac{1}{\rho_{d}}+\frac{2\rho_{d}^{2}\left(1-\gamma \right)\left(1-\gamma /2\right)}{1+\rho_{d}\left(1-2\gamma \right)+\rho_{d}^{2}{\left(1-\gamma \right)}^{2}}\right)$
${\bar{\Delta }}_{M/M/1/2^{*}}=\frac{1}{\mu }\left(1+\frac{1}{\rho }+\frac{\rho^{2}\left(1+3\rho +\rho^{2}\right)}{\left(1+\rho +\rho^{2}\right){\left(1+\rho \right)}^{2}}\right)$
${\bar{\Delta }}_{Ber/Geo/1/2^{*}}=\frac{1}{\gamma }\left(\left(1-\gamma \right)+\frac{1}{\rho_{d}}+\frac{\rho_{d}^{2}\left(1-\gamma \right)\left[1+3\rho_{d}\left(1-\gamma \right)+\rho_{d}^{2}\left(1-\gamma \right)\left(1-2\gamma \right)\right]}{\left[1+\rho_{d}\left(1-2\gamma \right)+\rho_{d}^{2}{\left(1-\gamma \right)}^{2}\right]{\left[1+\rho_{d}\left(1-\gamma \right)\right]}^{2}}\right)$
${\bar{\Delta }}_{M/M/1/\infty }=\frac{1}{\mu }\left(1+\frac{1}{\rho }+\frac{\rho^{2}}{1-\rho }\right)$
${\bar{\Delta }}_{Ber/Geo/1/\infty }=\frac{1}{\gamma }\left(\left(1-\gamma \right)+\frac{1}{\rho_{d}}+\frac{\rho_{d}^{2}\left(1-\gamma \right)}{1-\rho_{d}}\right)$

## Data Availability

Not applicable.

## Appendix A. Proof of Theorem 1

In this appendix, we derive all the results in Lemma 1 from the stationary Equations (18).

Define that

$$M_{1}=\sum_{n=1}^{\infty }\pi_{\left(n,0,0\right)},\;M_{2}=\sum_{m=1}^{\infty }\sum_{n=m+1}^{\infty }\pi_{\left(n,m,0\right)},\;\mathrm{and}\;M_{3}=\sum_{l=1}^{\infty }\sum_{m=l+1}^{\infty }\sum_{n=m+1}^{\infty }\pi_{\left(n,m,l\right)},$$

These three numbers are determined at the first place.

According to the last two rows of (18), we have

$$\begin{array}{cc} M_{1} & =\pi_{\left(1,0,0\right)}+\sum_{n=2}^{\infty }\pi_{\left(n,0,0\right)}\\ \; & =\left(\sum_{n=1}^{\infty }\pi_{\left(n,0,0\right)}\right)p\gamma +\sum_{n=2}^{\infty }\left\{\pi_{\left(n-1,0,0\right)}\left(1-p\right)+\sum_{k=n}^{\infty }\pi_{\left(k,n-1,0\right)}\left(1-p\right)\gamma \right\} \end{array}$$

(A1) $$\begin{array}{cc} \; & =p\gamma M_{1}+\left(1-p\right)M_{1}+\sum_{\tilde{m}=1}^{\infty }\sum_{\tilde{n}=\tilde{m}+1}^{\infty }\pi_{\left(\tilde{n},\tilde{m},0\right)}\left(1-p\right)\gamma \end{array}$$

(A2) $$\begin{array}{cc} \; & =p\gamma M_{1}+\left(1-p\right)M_{1}+\left(1-p\right)\gamma M_{2} \end{array}$$

where in (A1), we have used the substitutions $k=\tilde{n}$ and $n-1=\tilde{m}$. From Equation (A2), we obtain the first relation

(A3) $$p\left(1-\gamma \right)M_{1}=\left(1-p\right)\gamma M_{2}$$

Next, we deal with the number $M_{3}$ as follows.

$$\begin{array}{cc} M_{3} & =\sum_{l=1}^{\infty }\sum_{m=l+1}^{\infty }\sum_{n=m+1}^{\infty }\pi_{\left(n,m,l\right)} \end{array}$$

(A4) $$\begin{array}{c} =\sum_{m=2}^{\infty }\sum_{n=m+1}^{\infty }\pi_{\left(n,m,1\right)}+\sum_{l=2}^{\infty }\sum_{m=l+1}^{\infty }\sum_{n=m+1}^{\infty }\pi_{\left(n,m,l\right)} \end{array}$$

Using the first row of (18), the latter sum in Equation (A4) equals

(A5) $$\begin{array}{cc} \; & \sum_{l=2}^{\infty }\sum_{m=l+1}^{\infty }\sum_{n=m+1}^{\infty }\pi_{\left(n-1,m-1,l-1\right)}\left(1-\gamma \right)\left(1-p\eta \right)\\ = & \sum_{l=1}^{\infty }\sum_{m=l+1}^{\infty }\sum_{n=m+1}^{\infty }\pi_{\left(n,m,l\right)}\left(1-\gamma \right)\left(1-p\eta \right)\\ = & \left(1-\gamma \right)\left(1-p\eta \right)M_{3} \end{array}$$

and from the second and the third row of (18), the first part of (A4) is calculated as

$$\begin{array}{cc} \; & \sum_{n=3}^{\infty }\pi_{\left(n,2,1\right)}+\sum_{m=3}^{\infty }\sum_{n=m+1}^{\infty }\pi_{\left(n,m,1\right)}\\ = & \sum_{n=3}^{\infty }\pi_{\left(n-1,1,0\right)}p\left(1-\gamma \right)\\ & \;+\sum_{m=3}^{\infty }\sum_{n=m+1}^{\infty }\left\{\pi_{\left(n-1,m-1,0\right)}p\left(1-\gamma \right)+\sum_{j=1}^{m-2}\pi_{\left(n-1,m-1,j\right)}p\left(1-\gamma \right)\eta \right\}\\ = & \sum_{n=2}^{\infty }\pi_{\left(n,1,0\right)}p\left(1-\gamma \right)\\ & \;+\sum_{m=2}^{\infty }\sum_{n=m+1}^{\infty }\pi_{\left(n,m,0\right)}p\left(1-\gamma \right)+\sum_{m=3}^{\infty }\sum_{n=m+1}^{\infty }\sum_{j=1}^{m-2}\pi_{\left(n-1,m-1,j\right)}p\left(1-\gamma \right)\eta \end{array}$$

(A6) $$\begin{array}{cc} = & \sum_{m=1}^{\infty }\sum_{n=m+1}^{\infty }\pi_{\left(n,m,0\right)}p\left(1-\gamma \right)+\sum_{\tilde{m}=2}^{\infty }\sum_{\tilde{n}=\tilde{m}+1}^{\infty }\sum_{\tilde{l}=1}^{\tilde{m}-1}\pi_{\left(\tilde{n},\tilde{m},\tilde{l}\right)}p\left(1-\gamma \right)\eta \end{array}$$

(A7) $$\begin{array}{cc} = & p\left(1-\gamma \right)M_{2}+p\left(1-\gamma \right)\eta M_{3} \end{array}$$

where in (A6), we let $n-1=\tilde{n}$, $m-1=\tilde{m}$, and $j=\tilde{l}$. Equations (A4), (A5) and (A7) together give

(A8) $$p\left(1-\gamma \right)M_{2}=\gamma M_{3}$$

Since the sum of all the stationary probabilities equals 1, thus we have

(A9) $$M_{1}+M_{2}+M_{3}=1$$

Combining Equations (A3), (A8) and (A9), we can solve that

(A10) $$\begin{array}{cc} M_{1} & =\frac{\left(1-p\right)\gamma^{2}}{\left(p+\gamma -2p\gamma \right)\gamma +p^{2}{\left(1-\gamma \right)}^{2}} \end{array}$$

(A11) $$\begin{array}{cc} M_{2} & =\frac{p\gamma (1-\gamma )}{\left(p+\gamma -2p\gamma \right)\gamma +p^{2}{\left(1-\gamma \right)}^{2}} \end{array}$$

(A12) $$\begin{array}{cc} M_{3} & =\frac{p^{2}{\left(1-\gamma \right)}^{2}}{\left(p+\gamma -2p\gamma \right)\gamma +p^{2}{\left(1-\gamma \right)}^{2}} \end{array}$$

We mention that from the fourth, the fifth, and the sixth equation in (18), another relation can also be obtained for the second number $M_{2}$, which is given directly as

(A13) $$M_{2}=p\left(1-\gamma \right)M_{1}+p\gamma M_{2}+p\gamma \eta M_{3}+\left(1-p\right)\left(1-\gamma \right)M_{2}+\left(1-p\eta \right)\gamma M_{3}$$

which is reduced to (A8) when eliminating $M_{1}$ using the relation (A3).

Then, the relationships between functions $h_{i}\left(x\right)$, 1≤i≤3 and $h_{2}^{\left(m\right)}\left(x\right)$ are determined through similar procedures. First of all, we see that

$$\begin{array}{cc} h_{1}\left(x\right) & =\sum_{n=1}^{\infty }x^{n}\pi_{\left(n,0,0\right)}\\ \; & =x\pi_{\left(1,0,0\right)}+\sum_{n=2}^{\infty }x^{n}\left\{\pi_{\left(n-1,0,0\right)}\left(1-p\right)+\sum_{k=n}^{\infty }\pi_{\left(k,n-1,0\right)}\left(1-p\right)\gamma \right\}\\ \; & =p\gamma M_{1}x+\sum_{n=1}^{\infty }x^{n+1}\pi_{\left(n,0,0\right)}\left(1-p\right)+\sum_{n=2}^{\infty }x^{n}\sum_{k=n}^{\infty }\pi_{\left(k,n-1,0\right)}\left(1-p\right)\gamma \end{array}$$

(A14) $$\begin{array}{cc} \; & =p\gamma M_{1}x+\left(1-p\right)xh_{1}\left(x\right)+\sum_{\tilde{m}=1}^{\infty }x^{\tilde{m}+1}\sum_{\tilde{n}=\tilde{m}+1}^{\infty }\pi_{\left(\tilde{n},\tilde{m},0\right)}\left(1-p\right)\gamma \end{array}$$

(A15) $$\begin{array}{cc} \; & =p\gamma M_{1}x+\left(1-p\right)xh_{1}\left(x\right)+\left(1-p\right)\gamma xh_{2}^{\left(m\right)}\left(x\right) \end{array}$$

in which we denote $k=\tilde{n}$ and $n-1=\tilde{m}$.

From (A15), we obtain

(A16) $$h_{1}\left(x\right)=\frac{p\gamma M_{1}x}{1-(1-p)x}+\frac{(1-p)\gamma x}{1-(1-p)x}h_{2}^{\left(m\right)}\left(x\right)$$

Using stationary Equation (18), we determine function $h_{2}\left(x\right)$ in the following.

(A17) $$\begin{array}{cc} h_{2}\left(x\right) & =\sum_{m=1}^{\infty }\sum_{n=m+1}^{\infty }x^{n}\pi_{\left(n,m,0\right)}\\ \; & =\sum_{n=2}^{\infty }x^{n}\pi_{\left(n,1,0\right)}+\sum_{m=2}^{\infty }\sum_{n=m+1}^{\infty }x^{n}\\ \; & \;\times \left\{\pi_{\left(n-1,m-1,0\right)}\left(1-p\right)\left(1-\gamma \right)+\sum_{k=n}^{\infty }\pi_{\left(k,n-1,m-1\right)}\left(1-p\eta \right)\gamma \right\}\\ \; & =x^{2}\pi_{\left(2,1,0\right)}+\sum_{n=3}^{\infty }x^{n}\{\pi_{\left(n-1,0,0\right)}p\left(1-\gamma \right)+\sum_{k=n}^{\infty }\pi_{\left(k,n-1,0\right)}p\gamma \\ \; & \;+\sum_{k=n}^{\infty }\sum_{j=1}^{n-2}\pi_{\left(k,n-1,j\right)}p\gamma \eta \}+\sum_{m=1}^{\infty }\sum_{n=m+1}^{\infty }x^{n+1}\pi_{\left(n,m,0\right)}\left(1-p\right)\left(1-\gamma \right)\\ \; & \;+\sum_{m=2}^{\infty }\sum_{n=m+1}^{\infty }x^{n}\sum_{k=n}^{\infty }\pi_{\left(k,n-1,m-1\right)}\left(1-p\eta \right)\gamma \\ \; & =x^{2}\left\{\pi_{\left(1,0,0\right)}p\left(1-\gamma \right)+\sum_{k=2}^{\infty }\pi_{\left(k,1,0\right)}p\gamma \right\}+\sum_{n=2}^{\infty }x^{n+1}\pi_{\left(n,0,0\right)}p\left(1-\gamma \right)\\ \; & \;+\sum_{n=3}^{\infty }x^{n}\sum_{k=n}^{\infty }\pi_{\left(k,n-1,0\right)}p\gamma +\sum_{n=3}^{\infty }x^{n}\sum_{k=n}^{\infty }\sum_{j=1}^{n-2}\pi_{\left(k,n-1,j\right)}p\gamma \eta \\ \; & \;+\left(1-p\right)\left(1-\gamma \right)xh_{2}\left(x\right)+\left(1-p\eta \right)\gamma xh_{3}^{\left(m\right)}\left(x\right) \end{array}$$

Let $k=\tilde{n}$, $n-1=\tilde{m}$, and $m-1=\tilde{l}$, we have

(A18) $$\begin{array}{cc} \; & \sum_{m=2}^{\infty }\sum_{n=m+1}^{\infty }x^{n}\sum_{k=n}^{\infty }\pi_{\left(k,n-1,m-1\right)}\left(1-p\eta \right)\gamma \\ = & \sum_{\tilde{l}=1}^{\infty }\sum_{\tilde{m}=\tilde{l}+1}^{\infty }x^{\tilde{m}+1}\sum_{\tilde{n}=\tilde{m}+1}^{\infty }\pi_{\left(\tilde{n},\tilde{m},\tilde{l}\right)}\left(1-p\eta \right)\gamma \\ = & \left(1-p\eta \right)\gamma xh_{3}^{\left(m\right)}\left(x\right) \end{array}$$

where we define

(A19) $$h_{3}^{\left(m\right)}\left(x\right)=\sum_{l=1}^{\infty }\sum_{m=l+1}^{\infty }\sum_{n=m+1}^{\infty }x^{m}\pi_{\left(n,m,l\right)}$$

and the last term in Equation (A17) is obtained.

Continuing the calculation of (A17), we obtain that

(A20) $$\begin{array}{cc} h_{2}\left(x\right) & =p\left(1-\gamma \right)xh_{1}\left(x\right)+p\gamma xh_{2}^{\left(m\right)}\left(x\right)+\sum_{\tilde{m}=2}^{\infty }x^{\tilde{m}+1}\sum_{\tilde{n}=\tilde{m}+1}^{\infty }\sum_{\tilde{l}=1}^{\tilde{m}-1}\pi_{\left(\tilde{n},\tilde{m},\tilde{l}\right)}p\gamma \eta \\ \; & \;+\left(1-p\right)\left(1-\gamma \right)xh_{2}\left(x\right)+\left(1-p\eta \right)\gamma xh_{3}^{\left(m\right)}\left(x\right) \end{array}$$

In (A20), we have used the substitutions $k=\tilde{n}$, $n-1=\tilde{m}$, and $j=\tilde{l}$.

Except for the factor pγηx, the sum in (A20) is equal to

(A21) $$\begin{array}{cc} \; & x^{2}\sum_{n=3}^{\infty }\pi_{\left(n,2,1\right)}+x^{3}\sum_{n=4}^{\infty }\left[\pi_{\left(n,3,1\right)}+\pi_{\left(n,3,2\right)}\right]+x^{4}\sum_{n=5}^{\infty }\left[\pi_{\left(n,4,1\right)}+\pi_{\left(n,4,2\right)}+\pi_{\left(n,4,3\right)}\right]+\cdots \\ = & \sum_{m=2}^{\infty }x^{m}\sum_{n=m+1}^{\infty }\pi_{\left(n,m,1\right)}+\sum_{m=3}^{\infty }x^{m}\sum_{n=m+1}^{\infty }\pi_{\left(n,m,2\right)}+\sum_{m=4}^{\infty }x^{m}\sum_{n=m+1}^{\infty }\pi_{\left(n,m,3\right)}+\cdots \\ = & \sum_{l=1}^{\infty }\sum_{m=l+1}^{\infty }x^{m}\sum_{n=m+1}^{\infty }\pi_{\left(n,m,l\right)}\\ = & h_{3}^{\left(m\right)}\left(x\right) \end{array}$$

Substituting the result (A21) into Equation (A20) and merging the same terms gives

(A22) $$h_{2}\left(x\right)\left[1-(1-p)(1-\gamma )x\right]=p\left(1-\gamma \right)xh_{1}\left(x\right)+p\gamma xh_{2}^{\left(m\right)}\left(x\right)+\gamma xh_{3}^{\left(m\right)}\left(x\right)$$

We compute $h_{3}^{\left(m\right)}\left(x\right)$ while determining the other function $h_{2}^{\left(m\right)}\left(x\right)$ in the end. As before, from the equations in (18), we find that

(A23) $$\begin{array}{cc} h_{3}^{\left(m\right)}\left(x\right) & =\sum_{l=1}^{\infty }\sum_{m=l+1}^{\infty }\sum_{n=m+1}^{\infty }x^{m}\pi_{\left(n,m,l\right)}\\ \; & =\sum_{m=2}^{\infty }\sum_{n=m+1}^{\infty }x^{m}\pi_{\left(n,m,1\right)}+\sum_{l=2}^{\infty }\sum_{m=l+1}^{\infty }\sum_{n=m+1}^{\infty }x^{m}\pi_{\left(n-1,m-1,l-1\right)}\left(1-\gamma \right)\left(1-p\eta \right)\\ \; & =\sum_{n=3}^{\infty }x^{2}\pi_{\left(n,2,1\right)}+\sum_{m=3}^{\infty }\sum_{n=m+1}^{\infty }x^{m}\{\pi_{\left(n-1,m-1,0\right)}p\left(1-\gamma \right)\\ \; & \;+\sum_{j=1}^{m-2}\pi_{\left(n-1,m-1,j\right)}p\left(1-\gamma \right)\eta \}+\left(1-\gamma \right)\left(1-p\eta \right)xh_{3}^{\left(m\right)}\left(x\right)\\ \; & =\sum_{n=3}^{\infty }x^{2}\pi_{\left(n-1,1,0\right)}p\left(1-\gamma \right)+\sum_{m=2}^{\infty }\sum_{n=m+1}^{\infty }x^{m+1}\pi_{\left(n,m,0\right)}p\left(1-\gamma \right)\\ \; & \;+\sum_{m=3}^{\infty }\sum_{n=m+1}^{\infty }x^{m}\sum_{j=1}^{m-2}\pi_{\left(n-1,m-1,j\right)}p\left(1-\gamma \right)\eta +\left(1-\gamma \right)\left(1-p\eta \right)xh_{3}^{\left(m\right)}\left(x\right)\\ \; & =p\left(1-\gamma \right)xh_{2}^{\left(m\right)}\left(x\right)+p\left(1-\gamma \right)\eta xh_{3}^{\left(m\right)}\left(x\right)+\left(1-\gamma \right)\left(1-p\eta \right)xh_{3}^{\left(m\right)}\left(x\right)\\ \; & =p\left(1-\gamma \right)xh_{2}^{\left(m\right)}\left(x\right)+\left(1-\gamma \right)xh_{3}^{\left(m\right)}\left(x\right) \end{array}$$

which shows that

(A24) $$h_{3}^{\left(m\right)}\left(x\right)=\frac{p(1-\gamma )x}{1-(1-\gamma )x}h_{2}^{\left(m\right)}\left(x\right)$$

Combining Equations (A22) and (A24) yields the relation

(A25) $$h_{2}\left(x\right)=\frac{p(1-\gamma )x}{1-(1-p)(1-\gamma )x}h_{1}\left(x\right)+\frac{p\gamma x}{\left[1-(1-p)(1-\gamma )x][1-(1-\gamma )x\right]}h_{2}^{\left(m\right)}\left(x\right)$$

Now, we deal with function $h_{3}\left(x\right)$. Similarly to the process of obtaining (A23), we have

(A26) $$\begin{array}{cc} h_{3}\left(x\right) & =\sum_{m=2}^{\infty }\sum_{n=m+1}^{\infty }x^{n}\pi_{\left(n,m,1\right)}+\sum_{l=2}^{\infty }\sum_{m=l+1}^{\infty }\sum_{n=m+1}^{\infty }x^{n}\pi_{\left(n-1,m-1,l-1\right)}\left(1-\gamma \right)\left(1-p\eta \right)\\ \; & =\sum_{n=3}^{\infty }x^{n}\pi_{\left(n,2,1\right)}+\sum_{m=3}^{\infty }\sum_{n=m+1}^{\infty }x^{n}\{\pi_{\left(n-1,m-1,0\right)}p\left(1-\gamma \right)\\ \; & \;+\sum_{j=1}^{m-2}\pi_{\left(n-1,m-1,j\right)}p\left(1-\gamma \right)\eta \}+\left(1-\gamma \right)\left(1-p\eta \right)xh_{3}\left(x\right)\\ \; & =\sum_{n=3}^{\infty }x^{n}\pi_{\left(n-1,1,0\right)}p\left(1-\gamma \right)+\sum_{m=2}^{\infty }\sum_{n=m+1}^{\infty }x^{n+1}\pi_{\left(n,m,0\right)}p\left(1-\gamma \right)\\ \; & \;+\sum_{m=3}^{\infty }\sum_{n=m+1}^{\infty }x^{n}\sum_{j=1}^{m-2}\pi_{\left(n-1,m-1,j\right)}p\left(1-\gamma \right)\eta +\left(1-\gamma \right)\left(1-p\eta \right)xh_{3}\left(x\right)\\ \; & =p\left(1-\gamma \right)xh_{2}\left(x\right)+p\left(1-\gamma \right)\eta xh_{3}\left(x\right)+\left(1-\gamma \right)\left(1-p\eta \right)xh_{3}\left(x\right)\\ \; & =p\left(1-\gamma \right)xh_{2}\left(x\right)+\left(1-\gamma \right)xh_{3}\left(x\right) \end{array}$$

from which we derive

(A27) $$h_{3}\left(x\right)=\frac{p(1-\gamma )x}{1-(1-\gamma )x}h_{2}\left(x\right)$$

So far, we have obtained the relations (26)–(28) in Equations (A16), (A25) and (A27). To complete the proof of Lemma 1, the remaining part is the determination of the last function $h_{2}^{\left(m\right)}\left(x\right)$. It is shown that

(A28) $$\begin{array}{cc} h_{2}^{\left(m\right)}\left(x\right) & =\sum_{m=1}^{\infty }\sum_{n=m+1}^{\infty }x^{m}\pi_{\left(n,m,0\right)}\\ \; & =\sum_{n=2}^{\infty }x\pi_{\left(n,1,0\right)}+\sum_{m=2}^{\infty }\sum_{n=m+1}^{\infty }x^{m}\\ \; & \;\times \left\{\pi_{\left(n-1,m-1,0\right)}\left(1-p\right)\left(1-\gamma \right)+\sum_{k=n}^{\infty }\pi_{\left(k,n-1,m-1\right)}\left(1-p\eta \right)\gamma \right\}\\ \; & =x\pi_{\left(2,1,0\right)}+\sum_{n=3}^{\infty }x\{\pi_{\left(n-1,0,0\right)}p\left(1-\gamma \right)+\sum_{k=n}^{\infty }\pi_{\left(k,n-1,0\right)}p\gamma \\ \; & \;+\sum_{k=n}^{\infty }\sum_{j=1}^{n-2}\pi_{\left(k,n-1,j\right)}p\gamma \eta \}+\sum_{m=1}^{\infty }\sum_{n=m+1}^{\infty }x^{m+1}\pi_{\left(n,m,0\right)}\left(1-p\right)\left(1-\gamma \right)\\ \; & \;+\sum_{m=2}^{\infty }\sum_{n=m+1}^{\infty }x^{m}\sum_{k=n}^{\infty }\pi_{\left(k,n-1,m-1\right)}\left(1-p\eta \right)\gamma \\ \; & =x\left\{\pi_{\left(1,0,0\right)}p\left(1-\gamma \right)+\sum_{k=2}^{\infty }\pi_{\left(k,1,0\right)}p\gamma \right\}+\sum_{n=2}^{\infty }x\pi_{\left(n,0,0\right)}p\left(1-\gamma \right)\\ \; & \;+\sum_{n=3}^{\infty }x\sum_{k=n}^{\infty }\pi_{\left(k,n-1,0\right)}p\gamma +\sum_{n=3}^{\infty }x\sum_{k=n}^{\infty }\sum_{j=1}^{n-2}\pi_{\left(k,n-1,j\right)}p\gamma \eta \\ \; & \;+\left(1-p\right)\left(1-\gamma \right)xh_{2}^{\left(m\right)}\left(x\right)+\left(1-p\eta \right)\gamma xh_{3}^{\left(l\right)}\left(x\right) \end{array}$$

Similarly, we use the substitutions $k=\tilde{n}$, $n-1=\tilde{m}$, and $m-1=\tilde{l}$, and we write

(A29) $$\begin{array}{cc} \; & \sum_{m=2}^{\infty }\sum_{n=m+1}^{\infty }x^{m}\sum_{k=n}^{\infty }\pi_{\left(k,n-1,m-1\right)}\left(1-p\eta \right)\gamma \\ = & \sum_{\tilde{l}=1}^{\infty }\sum_{\tilde{m}=\tilde{l}+1}^{\infty }x^{\tilde{l}+1}\sum_{\tilde{n}=\tilde{m}+1}^{\infty }\pi_{\left(\tilde{n},\tilde{m},\tilde{l}\right)}\left(1-p\eta \right)\gamma \\ = & \left(1-p\eta \right)\gamma xh_{3}^{\left(l\right)}\left(x\right) \end{array}$$

Thus, the last term in Equation (A28) is obtained, where $h_{3}^{\left(l\right)}\left(x\right)$ is defined and determined as

(A30) $$\begin{array}{cc} h_{3}^{\left(l\right)}\left(x\right) & =\sum_{l=1}^{\infty }\sum_{m=l+1}^{\infty }x^{l}\sum_{n=m+1}^{\infty }\pi_{\left(n,m,l\right)}\\ \; & =\sum_{m=2}^{\infty }x\sum_{n=m+1}^{\infty }\pi_{\left(n,m,1\right)}+\sum_{l=2}^{\infty }\sum_{m=l+1}^{\infty }x^{l}\sum_{n=m+1}^{\infty }\pi_{\left(n-1,m-1,l-1\right)}\left(1-\gamma \right)\left(1-p\eta \right)\\ \; & =x\sum_{n=3}^{\infty }\pi_{\left(n,2,1\right)}+\sum_{m=3}^{\infty }x\sum_{n=m+1}^{\infty }\{\pi_{\left(n-1,m-1,0\right)}p\left(1-\gamma \right)\\ \; & \;+\sum_{j=1}^{m-2}\pi_{\left(n-1,m-1,j\right)}p\left(1-\gamma \right)\eta \}+\left(1-\gamma \right)\left(1-p\eta \right)xh_{3}^{\left(l\right)}\left(x\right)\\ \; & =x\sum_{n=3}^{\infty }\pi_{\left(n-1,1,0\right)}p\left(1-\gamma \right)+\sum_{m=2}^{\infty }x\sum_{n=m+1}^{\infty }\pi_{\left(n,m,0\right)}p\left(1-\gamma \right)\\ \; & \;+\sum_{m=3}^{\infty }x\sum_{n=m+1}^{\infty }\sum_{j=1}^{m-2}\pi_{\left(n-1,m-1,j\right)}p\left(1-\gamma \right)\eta +\left(1-\gamma \right)\left(1-p\eta \right)xh_{3}^{\left(l\right)}\left(x\right)\\ \; & =p\left(1-\gamma \right)M_{2}x+p\left(1-\gamma \right)\eta M_{3}x+\left(1-\gamma \right)\left(1-p\eta \right)xh_{3}^{\left(l\right)}\left(x\right)\\ \; & =\left[\gamma +p(1-\gamma )\eta \right]M_{3}x+\left(1-\gamma \right)\left(1-p\eta \right)xh_{3}^{\left(l\right)}\left(x\right) \end{array}$$

In Equation (A30), we have used the relation (A8), which says $p\left(1-\gamma \right)M_{2}=\gamma M_{3}$. From (A30), we obtain the result

(A31) $$h_{3}^{\left(l\right)}\left(x\right)=\frac{\left[\gamma +p(1-\gamma )\eta \right]M_{3}x}{1-(1-\gamma )(1-p\eta )x}$$

Coming back to the calculation of function $h_{2}^{\left(m\right)}\left(x\right)$, Equation (A28) shows

(A32) $$\begin{array}{cc} h_{2}^{\left(m\right)}\left(x\right)\left[1-(1-p)(1-\gamma )x\right] & =p\left(1-\gamma \right)M_{1}x+p\gamma M_{2}x+p\gamma \eta M_{3}x+\left(1-p\eta \right)\gamma xh_{3}^{\left(l\right)}\left(x\right)\\ \; & =\left[\gamma +p^{2}\left(1-\gamma \right)\eta \right]M_{2}x+\left(1-p\eta \right)\gamma xh_{3}^{\left(l\right)}\left(x\right), \end{array}$$

in which we have used Equations (A3) and (A8) to replace numbers $M_{1}$ and $M_{3}$. Substituting expression (A31), after some extra operations, we determine that

(A33) $$h_{2}^{\left(m\right)}\left(x\right)=\frac{\left[\gamma +p^{2}\left(1-\gamma \right)\eta -\left(1-p\right)\left(1-\gamma \right)\left(1-p\eta \right)\gamma x\right]M_{2}x}{\left[1-(1-p)(1-\gamma )x][1-(1-\gamma )(1-p\eta )x\right]}$$

This eventually completes the proof of Lemma 1.

## Appendix B. Proof of Equations (41) and (42)

In Equation (33), we show that

(A34) $$\begin{array}{cc} H_{PP}\left(x\right) & =\frac{p\gamma M_{1}x\left[1-\left(1-p\right)\left(1-\gamma \right)x\right]}{\left[1-(1-p)x][1-(1-\gamma )x\right]}\\ \; & \;+\frac{\gamma x\left\{1-\left(1-p\right)\left[2\left(1-\gamma \right)+p\gamma \right]x+{\left(1-p\right)}^{2}{\left(1-\gamma \right)}^{2}x^{2}\right\}}{\left[1-\left(1-p\right)x\right]{\left[1-\left(1-\gamma \right)x\right]}^{2}}h_{2}^{\left(m\right)}\left(x\right) \end{array}$$

Equations (41) and (42) are obtained by decomposing each part of (A34). For the first part, we assume

(A35) $$\begin{array}{cc} \frac{1-(1-p)(1-\gamma )x}{\left[1-(1-p)x][1-(1-\gamma )x\right]} & =\frac{A}{1-(1-p)x}+\frac{B}{1-(1-\gamma )x}\\ \; & =\frac{(A+B)-[A(1-\gamma )+B(1-p)]x}{\left[1-(1-p)x][1-(1-\gamma )x\right]} \end{array}$$

and according to the coefficients of corresponding terms, we obtain

(A36) A+B=1,A(1−γ)+B(1−p)=(1−p)(1−γ)

which determine *A* and *B* as

(A37) $$A=\frac{(1-p)\gamma }{\gamma -p},\;\mathrm{and}\;B=\frac{p(1-\gamma )}{\gamma -p}$$

Therefore,

(A38) $$\frac{p\gamma M_{1}x\left[1-\left(1-p\right)\left(1-\gamma \right)x\right]}{\left[1-(1-p)x][1-(1-\gamma )x\right]}=\frac{p\gamma M_{1}x}{\gamma -p}\left(\frac{(1-p)\gamma }{1-(1-p)x}-\frac{p(1-\gamma )}{1-(1-\gamma )x}\right)$$

For the second part of expression (A34), let

$$\begin{array}{cc} \; & \frac{1-\left(1-p\right)\left[2\left(1-\gamma \right)+p\gamma \right]x+{\left(1-p\right)}^{2}{\left(1-\gamma \right)}^{2}x^{2}}{\left[1-\left(1-p\right)x\right]{\left[1-\left(1-\gamma \right)x\right]}^{2}}\\ = & \frac{A}{1-(1-p)x}+\frac{B}{1-(1-\gamma )x}+\frac{C}{{\left[1-\left(1-\gamma \right)x\right]}^{2}}\\ = & \frac{\left(A+B+C\right)-\left[\left(A+B\right)\left(1-\gamma \right)+A\left(1-\gamma \right)+B\left(1-p\right)+C\left(1-p\right)\right]x+c_{2}x^{2}}{\left[1-\left(1-p\right)x\right]{\left[1-\left(1-\gamma \right)x\right]}^{2}} \end{array}$$

in which

$$c_{2}=\left[A\left(1-\gamma \right)+B\left(1-p\right)\right]\left(1-\gamma \right)$$

Thus, we have

(A39) $$\left\{\begin{array}{cc} 1=A+B+C & \;\\ \left(1-p)[2(1-\gamma )+p\gamma ]=(A+B)(1-\gamma )+A(1-\gamma )+B(1-p)+C(1-p\right) & \;\\ {\left(1-p\right)}^{2}\left(1-\gamma \right)=A\left(1-\gamma \right)+B\left(1-p\right) & \; \end{array}\right.$$

Substituting the third relation and A+B=1−C into the second equation, we obtain

(A40) $$\left(1-p\right)\left[2\left(1-\gamma \right)+p\gamma \right]=\left(1-C\right)\left(1-\gamma \right)+{\left(1-p\right)}^{2}\left(1-\gamma \right)+C\left(1-p\right)$$

from which we can solve that C=p.

Then, according to the equations

$$A+B=1-p\;\;\mathrm{and}\;\;A\left(1-\gamma \right)+B\left(1-p\right)={\left(1-p\right)}^{2}\left(1-\gamma \right)$$

the other two numbers are obtained to be

(A41) $$A=\frac{{\left(1-p\right)}^{2}\gamma }{\gamma -p},\;B=-\frac{p(1-p)(1-\gamma )}{\gamma -p}$$

Thus, the factorization of the second part is obtained.

The last part—that is, the function $h_{2}^{\left(m\right)}\left(x\right)$—is dealt with similarly. Omitting the straight-forward calculations, we directly find that

(A42) $$h_{2}^{\left(m\right)}\left(x\right)=-\frac{\eta (1-p)(p+\gamma -p\gamma )}{\left(1-\eta )[1-(1-p)(1-\gamma )x\right]}+\frac{\left(1-p\eta )[\gamma +p(1-\gamma )\eta \right]}{\left(1-\eta )[1-(1-\gamma )(1-p\eta )x\right]}$$

Notice that (1−η) is contained in the denominator of fractions in Equation (A42); thus, η≠1. When η=1, Equation (29) shows that

(A43) $${\left.h_{2}^{\left(m\right)}\left(x\right)\right|}_{\eta =1}=\frac{\gamma +p^{2}\left(1-\gamma \right)-{\left(1-p\right)}^{2}\left(1-\gamma \right)\gamma x}{{\left[1-\left(1-p\right)\left(1-\gamma \right)x\right]}^{2}}$$

Summarizing the above results, both the Equations (41) and (42) are determined.

## Appendix C. Factorization of Last Part of Equation (42)

We write

(A44) $$\begin{array}{cc} \; & \frac{p\gamma M_{2}x^{2}}{{\left[1-\left(1-\gamma \right)x\right]}^{2}}\cdot \frac{\gamma +p^{2}\left(1-\gamma \right)-{\left(1-p\right)}^{2}\left(1-\gamma \right)\gamma x}{{\left[1-\left(1-p\right)\left(1-\gamma \right)x\right]}^{2}}\\ = & p\gamma M_{2}x^{2}\left(\frac{A}{1-(1-\gamma )x}+\frac{B}{{\left[1-\left(1-\gamma \right)x\right]}^{2}}+\frac{C}{1-(1-p)(1-\gamma )x}+\frac{D}{{\left[1-\left(1-p\right)\left(1-\gamma \right)x\right]}^{2}}\right)\\ = & p\gamma M_{2}x^{2}\left(\frac{(A+B)-A(1-\gamma )x}{{\left[1-\left(1-\gamma \right)x\right]}^{2}}+\frac{(C+D)-C(1-p)(1-\gamma )x}{{\left[1-\left(1-p\right)\left(1-\gamma \right)x\right]}^{2}}\right) \end{array}$$

Merging the terms in the bracket of (A44), according to corresponding coefficients, it is shown that

(A45) $$\left\{\begin{array}{c} A+B+C+D=\gamma +p^{2}\left(1-\gamma \right)\\ 2\left(A+B\right)\left(1-p\right)+2\left(C+D\right)+A+C\left(1-p\right)={\left(1-p\right)}^{2}\gamma \\ \left(A+B\right){\left(1-p\right)}^{2}+2A\left(1-p\right)+\left(C+D\right)+2C\left(1-p\right)=0\\ A(1-p)+C=0 \end{array}\right.$$

The last row of (A45) shows that C=−A(1−p), and using the first relationship, the second and the third row of Equation (A45) are equivalent to

(A46) $$\left\{\begin{array}{c} 2\left(1-p\right)\left[\gamma +p^{2}\left(1-\gamma \right)-\left(C+D\right)\right]+2\left(C+D\right)+A-A{\left(1-p\right)}^{2}={\left(1-p\right)}^{2}\gamma \\ {\left(1-p\right)}^{2}\left[\gamma +p^{2}\left(1-\gamma \right)-\left(C+D\right)\right]+2A\left(1-p\right)+\left(C+D\right)-2A{\left(1-p\right)}^{2}=0 \end{array}\right.$$

which gives

(A47) $$p\left(2-p\right)\left(C+D\right)=-2p\left(1-p\right)A-{\left(1-p\right)}^{2}\left[\gamma +p^{2}\left(1-\gamma \right)\right]$$

and

(A48) $$2p\left(C+D\right)=-p\left(2-p\right)A+{\left(1-p\right)}^{2}\gamma -2\left(1-p\right)\left[\gamma +p^{2}\left(1-\gamma \right)\right]$$

Combining Equations (A47) and (A48), the coefficient *A* is solved as

(A49) $$A=\frac{2-p}{p^{3}}\left({\left(1-p\right)}^{2}\gamma -\frac{2\left(1-p\right)[\gamma +p^{2}\left(1-\gamma \right)]}{2-p}\right)$$

and immediately

(A50) $$C=-A\left(1-p\right)=-\frac{\left(1-p)(2-p\right)}{p^{3}}\left({\left(1-p\right)}^{2}\gamma -\frac{2\left(1-p\right)[\gamma +p^{2}\left(1-\gamma \right)]}{2-p}\right)$$

Using Equation (A47), we have

(A51) $$\begin{array}{cc} D & =\frac{-2p\left(1-p\right)A-{\left(1-p\right)}^{2}\left[\gamma +p^{2}\left(1-\gamma \right)\right]}{p(2-p)}-C\\ \; & =\frac{-2p\left(1-p\right)A-{\left(1-p\right)}^{2}\left[\gamma +p^{2}\left(1-\gamma \right)\right]}{p(2-p)}+A\left(1-p\right)\\ \; & =\frac{p^{2}\left(1-p\right)A-{\left(1-p\right)}^{2}\left[\gamma +p^{2}\left(1-\gamma \right)\right]}{p(2-p)} \end{array}$$

and in the end, the last number *B* is determined by

(A52) $$B=[\gamma +p^{2}\left(1-\gamma \right)]-A-C-D$$

So far, all the coefficients are obtained and the decomposition is totally determined.
